# Elevated S-adenosylhomocysteine induces adipocyte dysfunction to promote alcohol-associated liver steatosis

**DOI:** 10.1038/s41598-021-94180-x

**Published:** 2021-07-19

**Authors:** Madan Kumar Arumugam, Srinivas Chava, Karuna Rasineni, Matthew C. Paal, Terrence M. Donohue, Natalia A. Osna, Kusum K. Kharbanda

**Affiliations:** 1 Research Service (151), Veterans Affairs Nebraska-Western Iowa Health Care System, 4101 Woolworth Avenue, Omaha, NE 68105 USA; 2 Department of Internal Medicine, University of Nebraska Medical Center, Omaha, NE 68198 USA; 3 Department of Biochemistry and Molecular Biology, University of Nebraska Medical Center, Omaha, NE 68198 USA

**Keywords:** Biochemistry, Gastroenterology

## Abstract

It has been previously shown that chronic ethanol administration-induced increase in adipose tissue lipolysis and reduction in the secretion of protective adipokines collectively contribute to alcohol-associated liver disease (ALD) pathogenesis. Further studies have revealed that increased adipose S-adenosylhomocysteine (SAH) levels generate methylation defects that promote lipolysis. Here, we hypothesized that increased intracellular SAH alone causes additional related pathological changes in adipose tissue as seen with alcohol administration. To test this, we used 3-deazaadenosine (DZA), which selectively elevates intracellular SAH levels by blocking its hydrolysis. Fully differentiated 3T3-L1 adipocytes were treated in vitro for 48 h with DZA and analysed for lipolysis, adipokine release and differentiation status. DZA treatment enhanced adipocyte lipolysis, as judged by lower levels of intracellular triglycerides, reduced lipid droplet sizes and higher levels of glycerol and free fatty acids released into the culture medium. These findings coincided with activation of both adipose triglyceride lipase and hormone sensitive lipase. DZA treatment also significantly reduced adipocyte differentiation factors, impaired adiponectin and leptin secretion but increased release of pro-inflammatory cytokines, IL-6, TNF and MCP-1. Together, our results demonstrate that elevation of intracellular SAH alone by DZA treatment of 3T3-L1 adipocytes induces lipolysis and dysregulates adipokine secretion. Selective elevation of intracellular SAH by DZA treatment mimics ethanol’s effects and induces adipose dysfunction. We conclude that alcohol-induced elevations in adipose SAH levels contribute to the pathogenesis and progression of ALD.

## Introduction

Adipocytes, the primary component of fat tissues, mainly serve as a depot to store triglycerides. These cells have recently emerged as key regulators of the immune system as well as in modulating metabolism and behaviour^1, 2^. Adipose tissue communicates with other tissues and organs, including the liver, to integrate total body lipid homeostasis by controlling both circulation of free fatty acid (FFA) levels and secreting a host of biologically active proteins collectively known as adipokines^3^. Under physiological conditions both lipid storage and release are coordinated and tightly regulated so that dietary lipids are stored in a well-fed state and released during fasting to supply energy to the rest of the body. Lipolysis is the process responsible for the catabolism of triglycerides (TG) which generate FFAs that are subsequently used as energy substrates, as essential precursors for lipid/membrane synthesis, or as mediators in cell signalling processes^4^. The regulation of TG storage and non-esterified fatty acid (NEFA) release by adipose tissue is perturbed particularly when the release of FFAs becomes dissociated from energy requirements in extra-adipose tissues. This leads to increased circulating FFA levels and its uptake for storage as TGs in organs/tissue such as the liver, leading to the development of hepatic steatosis^5^.

Chronic alcohol exposure is associated with increased oxidative stress, cell death and inflammatory response in the liver^6^. Alcohol consumption can also profoundly disturb the normal function of adipose tissue by inducing adipocyte lipolysis, reducing secretion of adipokines, and increasing release of pro-inflammatory mediators, all of which promote the pathogenesis of alcohol-associated liver disease (ALD)^7, 8^. Particularly relevant in the context of alcohol-associated fatty liver is the contribution of increased flow of FFAs to the liver from enhanced adipose tissue lipolysis^9^. Adiponectin and leptin are the key adipokines that modulate hepatic lipid homeostasis toward reduction of lipid content in the liver^10^. Alcohol exposure has been shown to decrease the secretion of these adipokines and promote the development of hepatic steatosis^10, 11^. Further studies have reported that chronic alcohol consumption results in impaired methionine metabolism in adipose tissue, characterized by increased S-adenosylhomocysteine (SAH) levels and a consequent decrease in the S-adenosylmethionine (SAM):SAH ratio^12^. This loss in the methylation potential has been shown to enhance hormone sensitive lipase (HSL) activation to promote lipolysis in adipose tissue^13, 14^.

Based on these findings, we hypothesized that increased intracellular SAH alone can cause pathological changes in the adipocyte, as seen with alcohol administration. To test this, we exposed cultured differentiated 3T3-L1 adipocytes to 3-deazaadenosine (DZA), which causes intracellular SAH accumulation by blocking the activity of S-adenosylhomocysteine hydrolase^15^. We then examined the mechanisms underlying the effect of increased intracellular SAH to adipocyte dysfunction and the potential role in the progression of alcohol-associated fatty liver disease.

## Results

### DZA stimulates lipolysis in adipocytes

**Figure 1:**
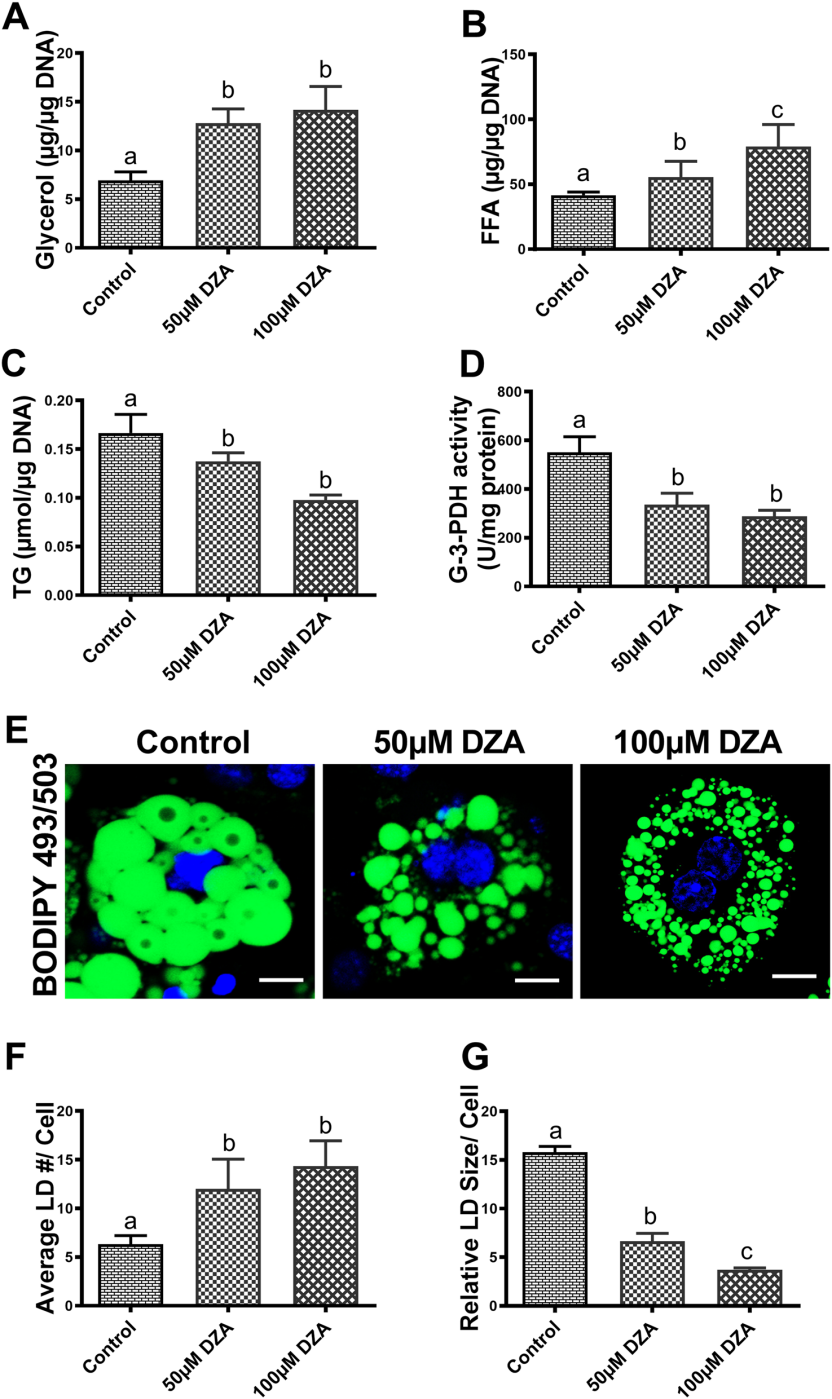
DZA stimulates lipolysis in 3T3-L1 adipocytes. Quantification of (**A**) glycerol and (**B**) free fatty acid (FFA) levels in the medium normalized to cellular DNA and expressed as µg/µg DNA, (**C**) cellular triglyceride (TG) content normalized to cellular DNA and expressed as µmole/µg DNA and (**D**) glycerol-3-phosphate dehydrogenase (G-3-PDH) activity expressed as U/mg protein (1 unit of activity represents the oxidation amount of 1.0 nmol/L per minute) in control and DZA-treated 3T3-L1 adipocytes (n = 8). (**E**) Representative confocal images of BODIPY 493/503 and DAPI (nucleus – blue) stained control and DZA-treated 3T3-L1 adipocytes (Scale bar—20 µm). Distribution of lipid droplets (LDs, stained green) in 3T3-L1 adipocytes, n = 4 experiments; images of 27 random cells from each treatment group per experiment were captured and analysed for (**F**) Average # of LDs per cell and (**G**) relative size (diameter) of LDs/cell. Data are presented as the mean ± SEM; values not sharing a common letter significantly differ from each other at *p* ≤ 0.05.

To examine whether DZA stimulates lipolysis, the differentiated 3T3-L1 adipocytes were treated with 0, 50 and 100 µM of DZA concentrations for 48 h. Hydrolysis of TG in adipocytes by cellular lipases results in the release of glycerol and FFAs^16^. Hence, their release in the incubation medium as well as total cellular TG content was examined to assess lipolysis^17^. DZA treatment for 48 h increased release of glycerol and FFAs into the medium by ~ 2 and 1.5-fold, respectively (Fig. 1A,B). In addition, we observed a parallel 1.5-fold decrease in the cellular content of TGs with 48 h DZA treatments (Fig. 1C). Taken together, these results indicate that DZA is the trigger that promotes active lipolysis in 3T3-L1 adipocytes. Next, we measured the activity of glycerol-3-phosphate dehydrogenase (G-3-PDH), an enzyme expressed in differentiated adipocytes and used to measure TG synthesis^18^. We observed 62% and 57% reduction in G-3-PDH enzyme following treatment with 50 and 100 µM DZA, respectively (Fig. 1D).

### Morphological assessment of adipocytes

There is a morphological and functional difference between 3T3-L1 preadipocytes and well-differentiated mature adipocytes. The first indication of 3T3-L1 preadipocyte differentiation into fully differentiated adipocytes is the accumulation of lipid droplets (LDs) which can be stained with BODIPY 493/503, a lipophilic fluorescent dye. BODIPY staining revealed characteristic larger sized intracellular LDs in control 3T3-L1 adipocytes, which became progressively smaller with increasing DZA treatments (Fig. 1E). Quantification analysis confirmed clear differences in LD numbers (Fig. 1F) and relative size (Fig. 1G) between the control and DZA-treated 3T3-L1 adipocytes. The widely distributed LDs in control 3T3-L1 adipocytes were larger in size (~ 15 µm in diameter) with fewer number of LDs per cell, whereas 50 and 100 µM DZA treated 3T3-L1 adipocytes showed higher numbers of much smaller-sized LDs (2–6 µm in diameter) per cell. This morphological assessment corroborated the biochemical assessment of lower cellular TG content and higher levels of glycerol and FFAs released into the medium portending that DZA treatments promote the hydrolysis of LD TG stores, thereby generating much smaller LDs.

### DZA treatment increases intracellular SAH and reduces methylation potential

**Figure 2:**
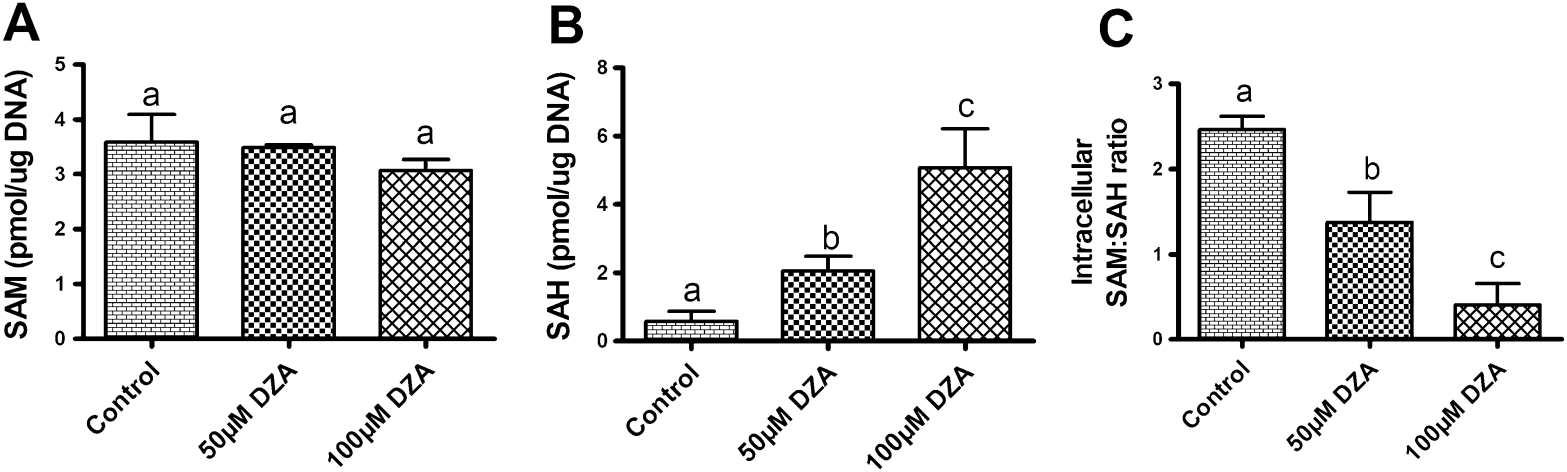
DZA reduces the methylation potential in 3T3-L1 adipocytes. (**A**) Intracellular SAM level, (**B**) SAH level and (**C**) Intracellular SAM:SAH ratio in control and DZA-treated 3T3-L1 adipocytes (n = 4). Data are presented as the mean ± SEM; values not sharing a common letter significantly differ from each other at *p* ≤ 0.05.

To determine the cellular methylation potential, intracellular SAM, SAH and SAM:SAH ratios were measured in mature adipocytes treated with different concentration of DZA. While DZA treatment did not affect intracellular SAM levels (Fig. 2A), it markedly increased SAH levels (Fig. 2B), resulting in a dose-dependent decrease in the SAM:SAH ratio (Fig. 2C).

### DZA activates ATGL in adipocytes

**Figure 3:**
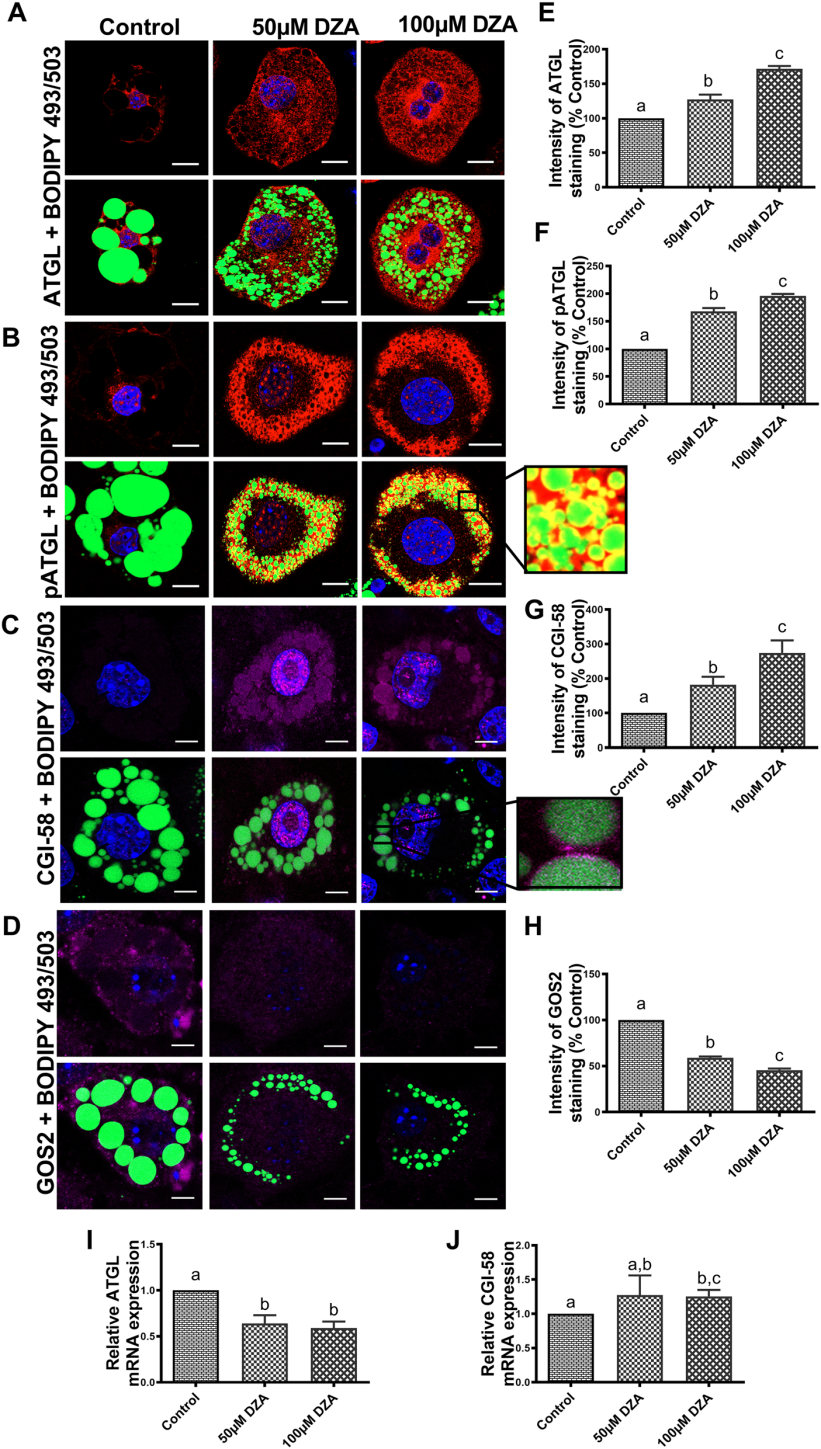
DZA activates ATGL in 3T3-L1 adipocytes. Immunofluorescence images showing localization of (**A**) ATGL, (**B**) pATGL-Ser(406), (**C**) CGI-58 and (**D**) GOS2 in BODIPY 493/503 and DAPI stained control and DZA-treated 3T3-L1 adipocytes (Scale bar—20 µm) with a magnified images of a representative 100 µM DZA-treated cell showing localization of pATGL-Ser(406) and CGI-58 on the LD surface. (**E**) The fluorescence intensity of ATGL, (**F**) pATGL-Ser(406), (**G**) CGI-58 and (**H**) GOS2 expression in each group of cells normalized to % of control cells, (n = 4) experiments; confocal images of 31 random cells from CGI-58 mRNA expression in control and DZA-treated 3T3-L1 adipocytes, n = 5. Data are presented as the mean ± SEM; values not sharing a common letter significantly differ from each other at *p* ≤ 0.05.

Adipose triglyceride lipase (ATGL) is the rate-limiting enzyme for TG hydrolysis in adipocytes^19^ and comparative gene identification-58 (CGI-58) is its coactivator^20, 21^. The protein coded by G0/G1 switch gene 2 (GOS2) is highly expressed in adipocytes and specifically interacts with ATGL inhibiting its TG hydrolase activity^19, 22–24^. Immunofluorescent staining (Fig. 3A–D) and quantitative analysis (Fig. 3E–H) revealed decreased GOS2 but increased ATGL, activated ATGL (pATGL-Ser(406)) and CGI-58 in DZA-treated 3T3-L1 adipocytes compared to control cells. BODIPY co-staining showed cellular localization of pATGL-Ser(406) at the LD surface of DZA-treated 3T3-L1 adipocytes compared to untreated control cells (Fig. 3B inset). Consistent with the protein expression data, RT-PCR revealed higher level of mRNA encoding for CGI-58 (Fig. 3J) in DZA-treated 3T3-L1 adipocytes compared with untreated adipocytes. However, despite an increase in overall ATGL protein expression, lower mRNA levels were observed in both DZA-treated 3T3-L1 adipocytes compared with untreated controls (Fig. 3I).

### DZA activates HSL in adipocytes

**Figure 4:**
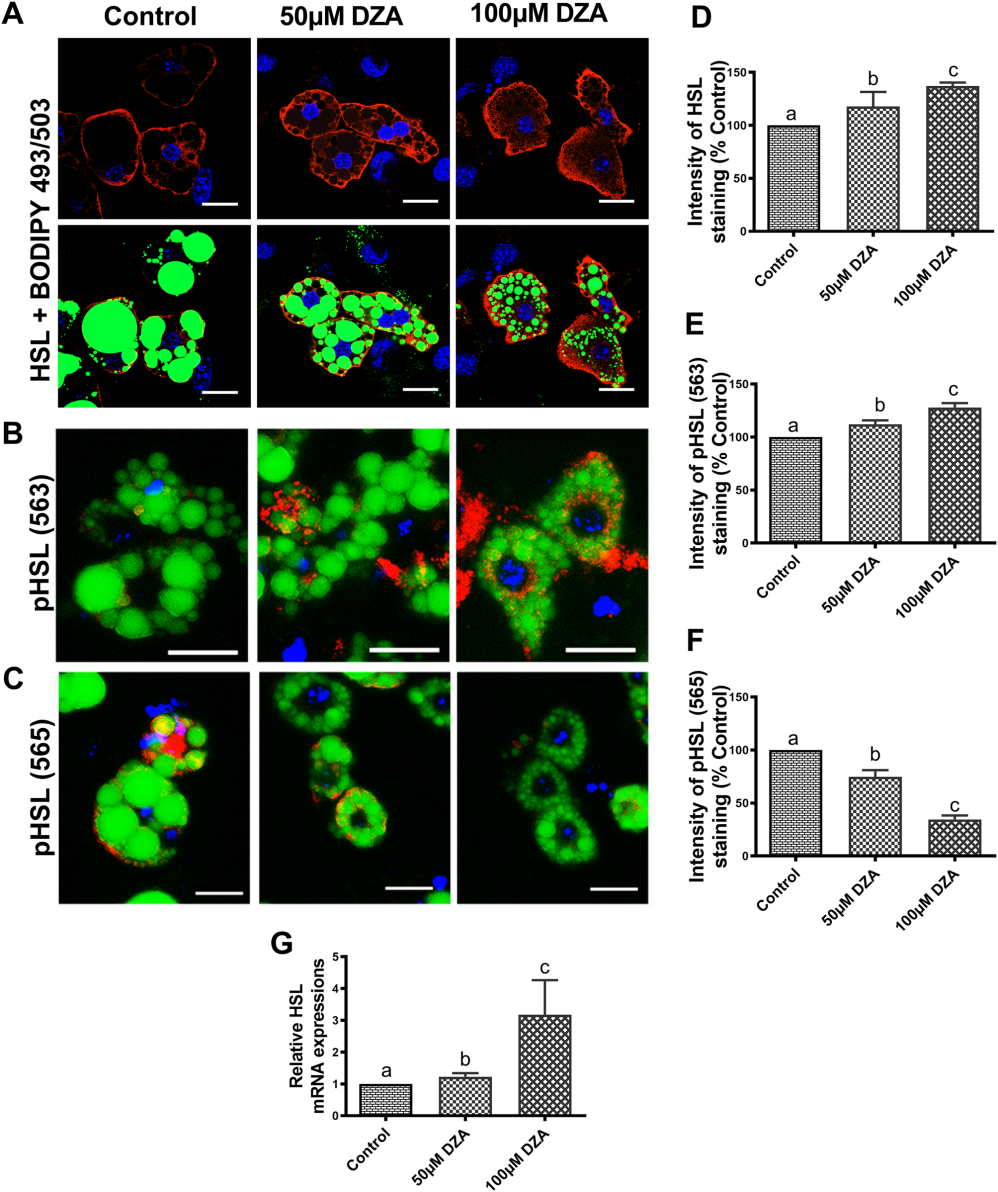
DZA activates HSL in 3T3-L1 adipocytes. Representative immunofluorescence images of (**A**) total HSL (**B**) pHSL-Ser(563), and (**C**) pHSL-Ser(565) in BODIPY 493/503 and DAPI stained control and DZA-treated 3T3-L1 adipocytes (Scale bar—20 µm). The fluorescence intensity of (**D**) HSL, (**E**) pHSL-Ser(563) and (**F**) pHSL-Ser(565) in DZA-treated cells normalized to % of control cells, n = 4 experiments; Images (confocal microscope—Panel A; Keyence BZ-X810 florescence microscope—Panels B and C) of 22 random cells from each treatment group per experiment were captured and analysed. (**G**) Relative HSL mRNA levels in control and DZA-treated 3T3-L1 adipocytes (n = 5). Data are presented as the mean ± SEM; values not sharing a common letter significantly differ from each other at *p* ≤ 0.05.

HSL, another important lipase, is actively involved in adipocyte lipolysis^25^. We sought to determine whether DZA influences HSL and the phosphorylated form of HSL in 3T3-L1 adipocytes. Immunofluorescent staining and RT-PCR analyses showed that DZA treatments increased total HSL protein (Fig. 4A,D) and its mRNA (Fig. 4G) compared to control cells. Phosphorylation at Ser-563 activates HSL facilitating its translocation from the cytosol to LDs while phosphorylation at Ser-565 has been suggested to have an antilipolytic role^26^. Immunofluorescence imagery and quantitative analysis of pHSL-Ser(563) and pHSL-Ser(565) expression revealed DZA-induced an increase in pHSL-Ser(563) and a concomitant decrease in pHSL-Ser(565) level compared to control cells (Fig. 4B,C,E,F).

### DZA modulates the expression of LD- and adipocyte-specific proteins

**Figure 5:**
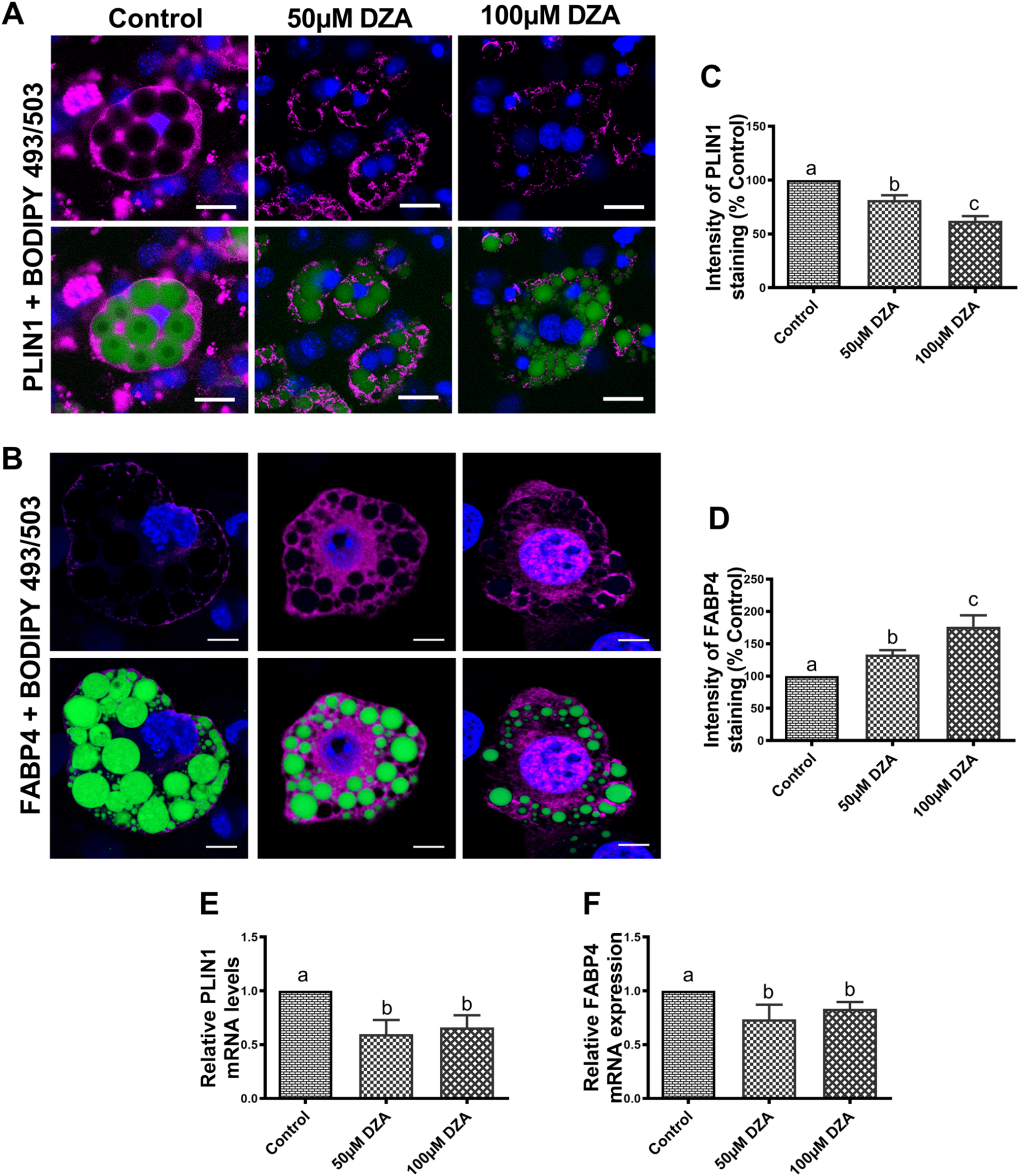
DZA modulates the expression of adipocyte-specific proteins. Representative immunofluorescence image of (**A**) PLIN1 and (**B**) FABP4 in BODIPY 493/503 and DAPI stained control and DZA-treated 3T3-L1 adipocytes (Scale bar—20 µm). (**C**) Fluorescence intensity of PLIN1 and (**D**) FABP4 in representative DZA-treated cells normalized to % of control cells, n = 4 experiments; images of 30 random cells from each treatment group per experiment were captured using Keyence BZ-X810 florescence microscope (Panel A) or confocal microscope (Panel B) and analysed. Relative mRNA expression levels of (**E**) PLIN1 and (**F**) FABP4 in control and DZA-treated 3T3-L1 adipocytes (n = 6). Data are presented as the mean ± SEM; values not sharing a common letter significantly differ from each other at *p* ≤ 0.05.

We then determined whether DZA modulates the expression of LD-associated proteins in adipocytes (Fig. 5A,B). Perilipin1 (PLIN1) is located on the surface of intracellular LDs and regulates lipolysis by inhibiting ATGL activity in adipocytes^27^. Fatty acid binding protein 4 (FABP4) is a fatty acid carrier protein primarily expressed in adipocytes^28^ that also regulates lipolysis^29^. Immunofluorescence image analysis showed lower PLIN1 but higher FABP4 expression on the surface of LDs stained with BODIPY 493/503 in DZA-treated 3T3-L1 adipocytes compared with controls (Fig. 5A–D). In accordance with the protein expression data, lower PLIN1 mRNA levels were observed in DZA-treated 3T3-L1 adipocytes compared with controls (Fig. 5E). However, despite an increase in overall FABP4 protein expression, lower mRNA levels were observed in both DZA-treated 3T3-L1 adipocytes compared with untreated controls (Fig. 5F).

### DZA downregulates protective adipokine secretion

**Figure 6:**
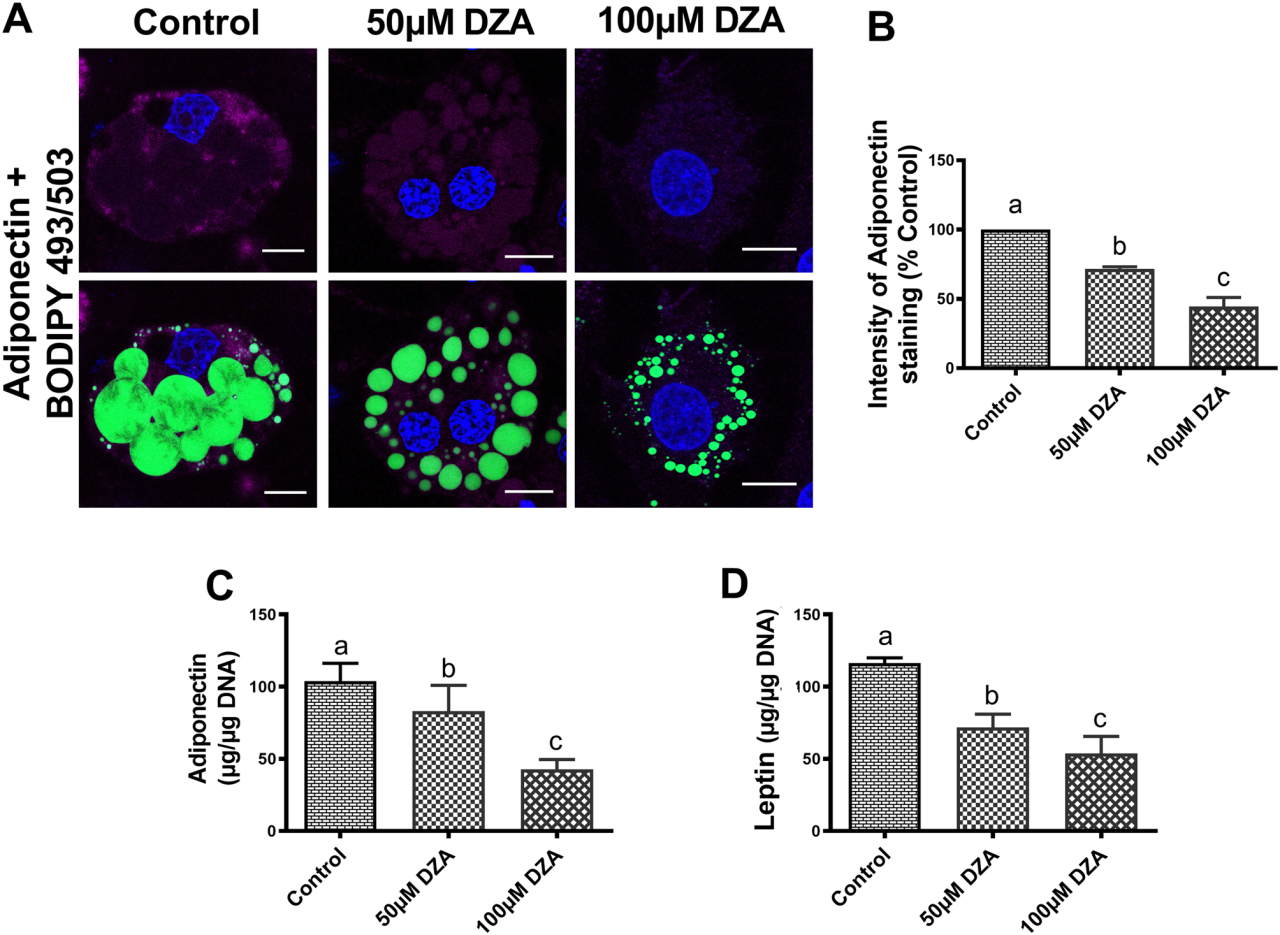
DZA downregulates protective adipokine secretion. Representative immunofluorescence image of (**A**) adiponectin in BODIPY 493/503 and DAPI stained control and DZA-treated 3T3-L1 adipocytes (Scale bar—20 µm). (**B**) Fluorescence intensity of adiponectin in representative DZA-treated cells normalized to % of control cells, n = 4 experiments; images of 32 random cells from each treatment group per experiment were captured using confocal microscope. (**C**) Adiponectin and (**D**) leptin levels in the culture medium of control and DZA-treated 3T3-L1 adipocytes normalized to cellular DNA and expressed as µg/µg DNA, n = 6. Data are presented as the mean ± SEM; values not sharing a common letter significantly differ from each other at *p* ≤ 0.05.

Immunofluorescent staining revealed a significant decrease in adiponectin protein expression and a concomitant decrease in its secretion in DZA-treated 3T3-L1 adipocytes compared with untreated control adipocytes (Fig. 6A–C). A similar dose-dependent reduction in secreted leptin levels were observed with DZA treatment of 3T3-L1 adipocytes compared with untreated control cells (Fig. 6D).

### DZA promotes the release of inflammatory cytokines from adipocytes

**Figure 7:**
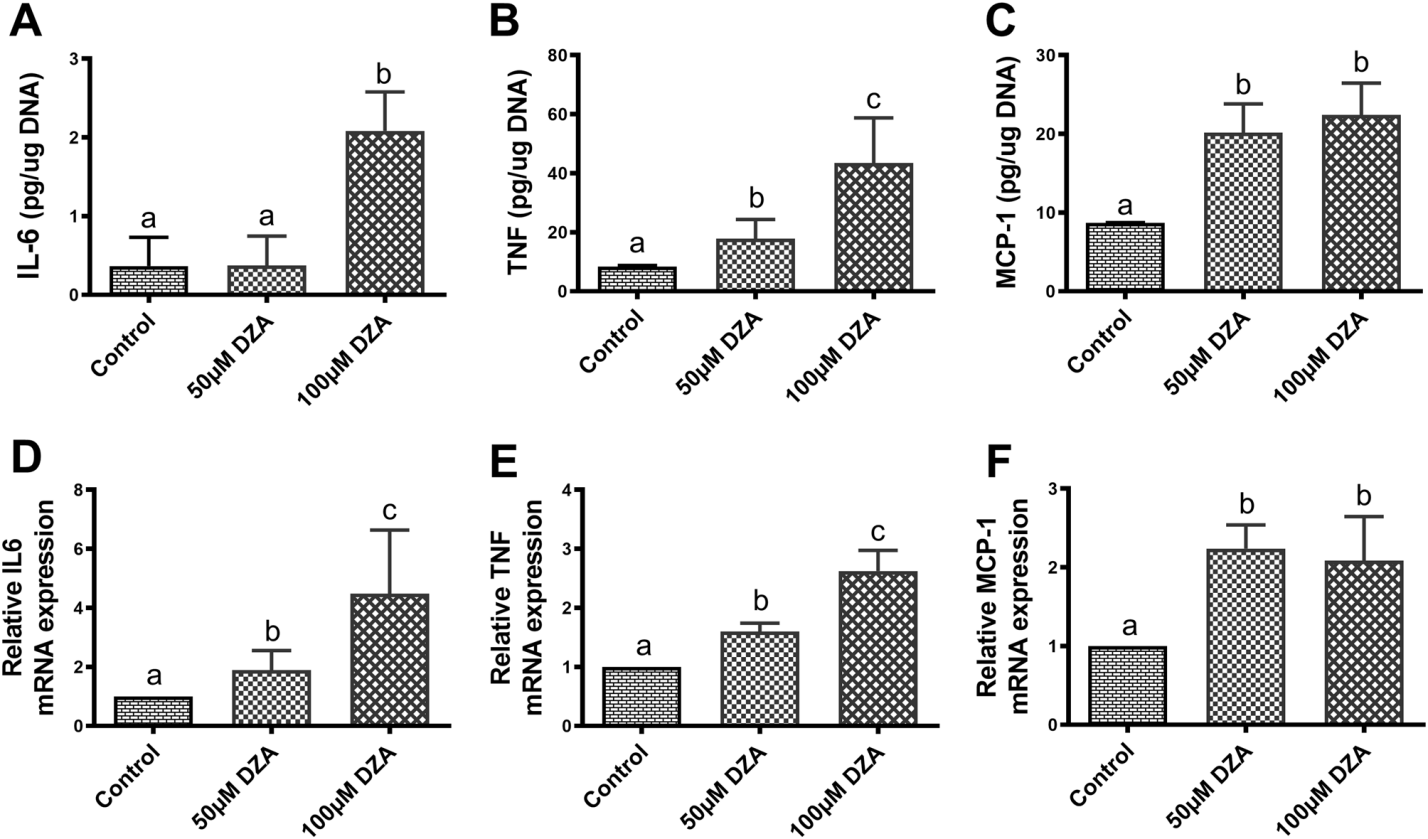
DZA promotes the release of inflammatory cytokines from 3T3-L1 adipocytes. Quantification of proinflammatory cytokines (**A**) IL-6, (**B**) TNF and (**C**) MCP-1 in the spent medium of control and DZA-treated 3T3-L1 adipocytes normalized to cellular DNA and expressed as µg/µg DNA (n = 5). Relative mRNA expression levels of (**D**) IL-6, (**E**) TNF and (**F**) MCP-1 in control and DZA-treated 3T3-L1 adipocytes (n = 5). Data are presented as the mean ± SEM; values not sharing a common letter significantly differ from each other at *p* ≤ 0.05.

Increased levels of secreted proinflammatory cytokines and chemokines act on adipocytes in a paracrine manner to promote their dysfunction^30^. We observed significantly higher levels of the inflammatory cytokines interleukin-6 (IL-6), tumour necrosis factor (TNF) and macrophage chemotactic protein-1 (MCP-1) and their mRNA levels (Fig. 7A–F) in DZA-treated 3T3-L1 adipocytes compared with untreated control adipocytes.

### DZA alters the expression of adipocyte differentiation markers

**Figure 8:**
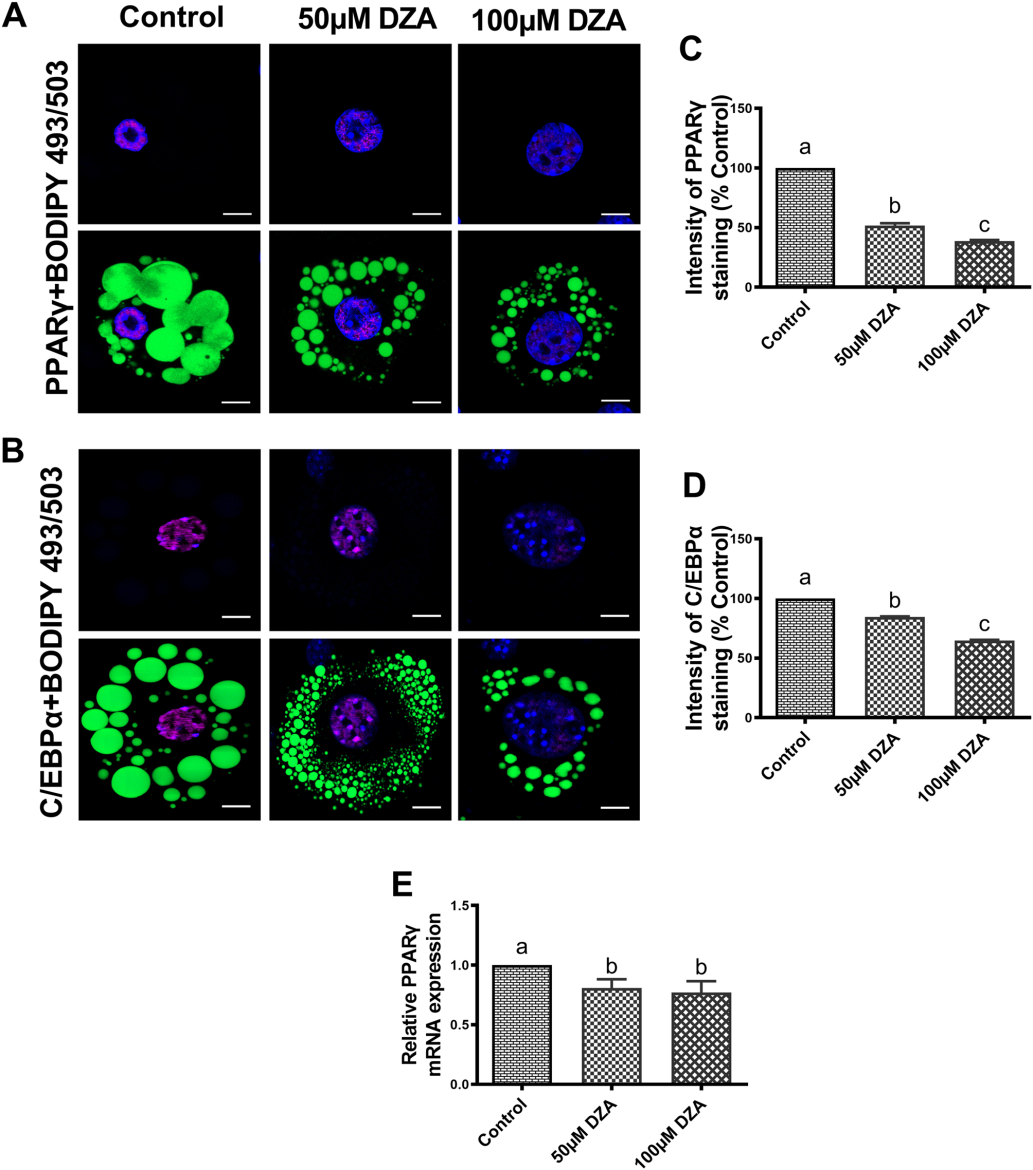
DZA alters the expression of adipocyte differentiation markers. (**A**) Representative immunofluorescence image of (**A**) PPARγ and (**B**) C/EBPα in BODIPY 493/503 and DAPI stained control and DZA-treated 3T3-L1 adipocytes (Scale bar—20 µm) and fluorescence intensity of (**C**) PPARγ and (**D**) C/EBPα in representative DZA-treated cells normalized to % of control cells, n = 5 experiments; images of 32 random cells from each treatment group per experiment were captured using confocal microscope. (**E**) Relative mRNA expression levels of PPARγ in control and DZA-treated 3T3-L1 adipocytes (n = 6). Data are presented as the mean ± SEM; values not sharing a common letter significantly differ from each other at *p* ≤ 0.05.

Adipocyte differentiation is controlled by a transcriptional cascade composed of key players such as peroxisome proliferator-activated receptor gamma (PPARγ) and CCAAT/enhancer-binding protein alpha (C/EBPα)^31^. We next investigated whether DZA affects 3T3-L1 adipocyte differentiation by conducting immunofluorescent staining and RT-PCR analyses. DZA exposure downregulated the protein expression of PPARγ and C/EBPα (Fig. 8A–C) and PPARγ mRNA expression (Fig. 8D) compared with untreated control 3T3-L1 adipocytes.

## Discussion

This present study reveals that DZA treatment selectively elevates intracellular SAH levels, increases lipolysis and dysregulates adipokine secretion, thus mimicking the effects of chronic ethanol consumption on adipose tissue as seen in experimental rodent models^9, 32–34^. DZA’s effects on adipocyte TG lipolysis occurred largely by (1) enhancing the activation of two key lipases, ATGL and HSL, (2) decreasing TG biosynthesis, (3) lowering the level of anti-lipolytic LD-associated protein, PLIN1 and (4) increasing the pro-lipolytic LD-associated proteins, CGI-58 and FABP4. Further, DZA treatment resulted in a decrease in the secretion of protective adipokines, adiponectin and leptin, while increasing the release of pro-inflammatory mediators, TNFα, MCP-1 and IL-6. The net effect of all these SAH-induced changes in adipocytes are known to play a causal role in the pathogenesis and progression of ALD^5, 7–9, 12, 32–36^.

We have maintained a long-standing interest in examining the alcohol-induced alterations in the methionine metabolic pathway and its functional ramifications. We have previously reported that the alcohol-induced increases in hepatocellular SAH and the resultant lowering of SAM:SAH ratio lead to the development of ALD features, including hepatic steatosis^37–40^. In expanding our examination of this pathway in other tissues and organs, we recently confirmed that chronic ethanol administration results in a similar reduction in the intracellular SAM:SAH ratio in epididymal white adipose tissue (eWAT; Supplementary Fig. S1A) as seen in the liver^37–40^. This hypomethylation status in eWAT is associated with (1) a reduced size of epididymal adipocytes reflecting a reduction in the size of the unilocular LD (Supplementary Figs. S1B & S1C), as also reported by others^32, 34^, (2) an increased activation of ATGL and HSL (Supplementary Fig. S1D), (3) increased serum NEFA level (Supplementary Fig. S2). These alcohol-induced changes in WAT with concurrent increases in liver fatty acid transport/binding proteins (cluster of differentiation 36 (CD36; Supplementary Fig. S3A) and adipose-specific FABP4 (Supplementary Fig. S3B)) facilitate increased uptake of adipose-derived circulating FFA, thereby contributing to the development of hepatic steatosis (Supplementary Figs. S3C & S3D). Here, we sought to examine the role of alcohol-induced hypomethylation state of eWAT to the rise in lipolysis and modulation in adipokine release by inducing intracellular SAH accumulation in differentiated 3T3-L1 adipocytes by DZA and examining whether this treatment alone could mimic the pathological changes as seen in vivo after alcohol administration. Specifically, we focused our investigation on those factors that contribute to the progression of alcohol-associated fatty liver disease^7, 8, 32–34^.

Here, we observed that the rise in intracellular SAH and the reduction in the methylation potential in adipocytes, caused by 48 h DZA treatment, enhanced the release of glycerol and FFAs into the spent medium, indicating a rise in lipolysis, as was previously reported^12^. We also observed that DZA treatment reduced intracellular TG levels in adipocytes, which could not only result from enhanced lipolysis as seen in these cells but also from impaired synthesis. Thus, we examined G-3-PDH, an important enzyme in the TG biosynthetic pathway, which is highly expressed in differentiating adipocytes^41^ and catalyses the reversible conversion between dihydroxyacetone phosphate and glycerol-3-phosphate^42^. We observed that the activity of G-3-PDH is reduced indicating that DZA also inhibits TG biosynthesis in adipocytes. DZA treatment also inhibited the de novo lipogenesis as shown by a significant decline in acetyl-CoA carboxylase (ACC) and fatty acid synthase (FAS) levels in adipocytes (Supplementary Figs. S4A-C). Thus, both enhanced lipolysis and reduced biosynthesis contribute to a loss in the cellular TGs observed. This decrease in cellular TGs by DZA in adipocytes is also reflected by a reduction in LD size as visualized by BODIPY 493/503 staining.

Stored cellular TGs are hydrolysed to glycerol and FFA in a three-step process catalysed by the consecutive action of ATGL, HSL, and monoglyceride lipase^4, 43, 44^. Based on an increase in lipolysis in adipocytes by DZA treatments, we then concentrated on the lipases and their activation status in these cells. ATGL is the key lipase, which hydrolyse TG to fatty acids^45^ and its elevated expression in 3T3-L1 adipocytes has been shown to increase both glycerol and FFA release^46^. CGI-58 is a coactivator of ATGL^20, 47^ and accelerates TG hydrolysis^21^, whereas GOS2 is a potent endogenous peptide inhibitor of ATGL and its hydrolase function ^19, 22–24^. ATGL and CGI-58 form a complex that catalyses the initial steps of lipolysis at the surface of the LD^24, 48^. In this study, we observed decreased GOS2 levels in conjunction with increased expression of total ATGL, its activated form and CGI-58 after DZA treatments indicating that this lipase is contributing to the increased lipolysis seen in 3T3-L1 adipocytes. Surprisingly, despite increased ATGL and FABP4 protein expression, their mRNA levels were lowered by DZA treatments. We are currently investigating the mechanism for the discordance between these proteins and their mRNA levels as has previously been reported in other studies^49–52^.

HSL is another major rate determining lipase in adipocyte lipolysis^25^. The activation and phosphorylation of HSL allows its translocation from cytosol to the LD surface, where it stimulates lipolysis^53^. HSL activity is post-translationally regulated by phosphorylation at multiple serine residues, in which Ser(563) is considered to be one of the major phosphorylation sites associated with increased HSL activity^54^. On the other hand, HSL activity is negatively regulated by phosphorylation at Ser(565)^55^, as it impairs phosphorylation on Ser(563) to decrease HSL activity^26, 55, 56^. Here, we observed that DZA treatment increased total HSL and pHSL-Ser(563) levels but decreased pHSL-Ser(565) level in 3T3-L1 adipocytes indicating that this lipase is also activated.

PLIN1, the best-known member of the PLIN family of proteins, is the predominant isoform found on the LD surface of mature adipocytes^16^. PLIN1 modulates lipolysis by regulating the interaction of ATGL with its coactivator CGI-58^57, 58^, and also by limiting the access of cytosolic lipases to the LDs, thereby facilitating TG storage under basal conditions^59^. Following a lipolytic trigger, PLIN1 gets phosphorylated which not only dissociates CGI-58, leading to ATGL activation but also helps recruit phosphorylated HSL to the LD surface^16, 24, 57, 60^. FABP4, a member of the cytosolic fatty acid binding protein family that it highly expressed in adipocytes^61^, plays a role in transporting FFAs to subcellular compartments^16^. It has been shown that FABP4 physically interacts with HSL to increase its lipolytic activity and can also form a complex with CGI-58 to increase hydrolysis of LD TG stores ^62–64^. In this study, we found decreased PLIN1 and increased FABP4 and CGI-58 expression in DZA-treated adipocytes, which together promote the activation of both lipases, HSL and ATGL, and their access to LD to ultimately enhance lipolysis in these cells.

Adipose dysfunction is characterized by proinflammatory macrophage polarization and altered adipokine secretion^65^. The adipokines, adiponectin and leptin, are secreted almost exclusively by adipocytes and reduction in their serum levels are implicated in the development of alcohol-associated fatty liver disease^11, 13, 33, 35, 36, 66^. In this study, we found that adipocytes treated with DZA secrete much less adiponectin and leptin compared to control adipocytes. Interestingly, we also observed a modest decrease in cellular adiponectin content. The cellular decrease in adiponectin by DZA treatment was much less in magnitude than that secreted, which showed a decline of almost 50% with the higher DZA dose compared with control. These data indicated that DZA may have a greater effect in disrupting intracellular trafficking of adiponectin as documented in a previous study which showed a 40% reduction in its secretion from subcutaneous adipocytes of rats fed ethanol compared with pair-fed controls^33^.

Here, we found that DZA treatment enhanced the release of inflammatory cytokines by 3T3-L1 adipocytes including TNF, IL-6 and MCP-1. Such increases have been reported in adipose depots of ethanol-fed rats but have been attributed to infiltrating macrophages and other immune cells^32^. Here we show that adipocytes also can secrete these cytokines as has been reported before by other investigators^67–69^. DZA treatment-induced increased lipolysis could also contribute to the production of these cytokines as shown by many investigators^70–72^.

Adipogenesis is regulated by a complex mechanism including transcriptional factors such as PPARγ and C/EBPα^73^. PPARγ is highly expressed in adipocytes, where it plays an essential role in the differentiation by regulating the expressions of genes responsible for maturation of the adipocyte phenotype^73, 74^. PPARγ cooperates with C/EBPα to regulate adipocyte differentiation and induces the expression of FABP4^75^. In the present study DZA suppressed adipogenesis by downregulating PPARγ and C/EBPα in addition to upregulating major lipases to induce lipolysis, reducing TG synthesis and suppressing both cellular and secretory levels of adiponectin. All these changes could be reflective of a de-differentiation of 3T3-L1 adipocytes by DZA. However, we observed an increased release of pro-inflammatory cytokines, IL-6, TNF and MCP-1, and an increase in differentiated adipocyte-specific binding protein (FABP4) with DZA treatment suggesting that it is inducing adipocyte dysfunction, rather than de-differentiation.

**Figure 9:**
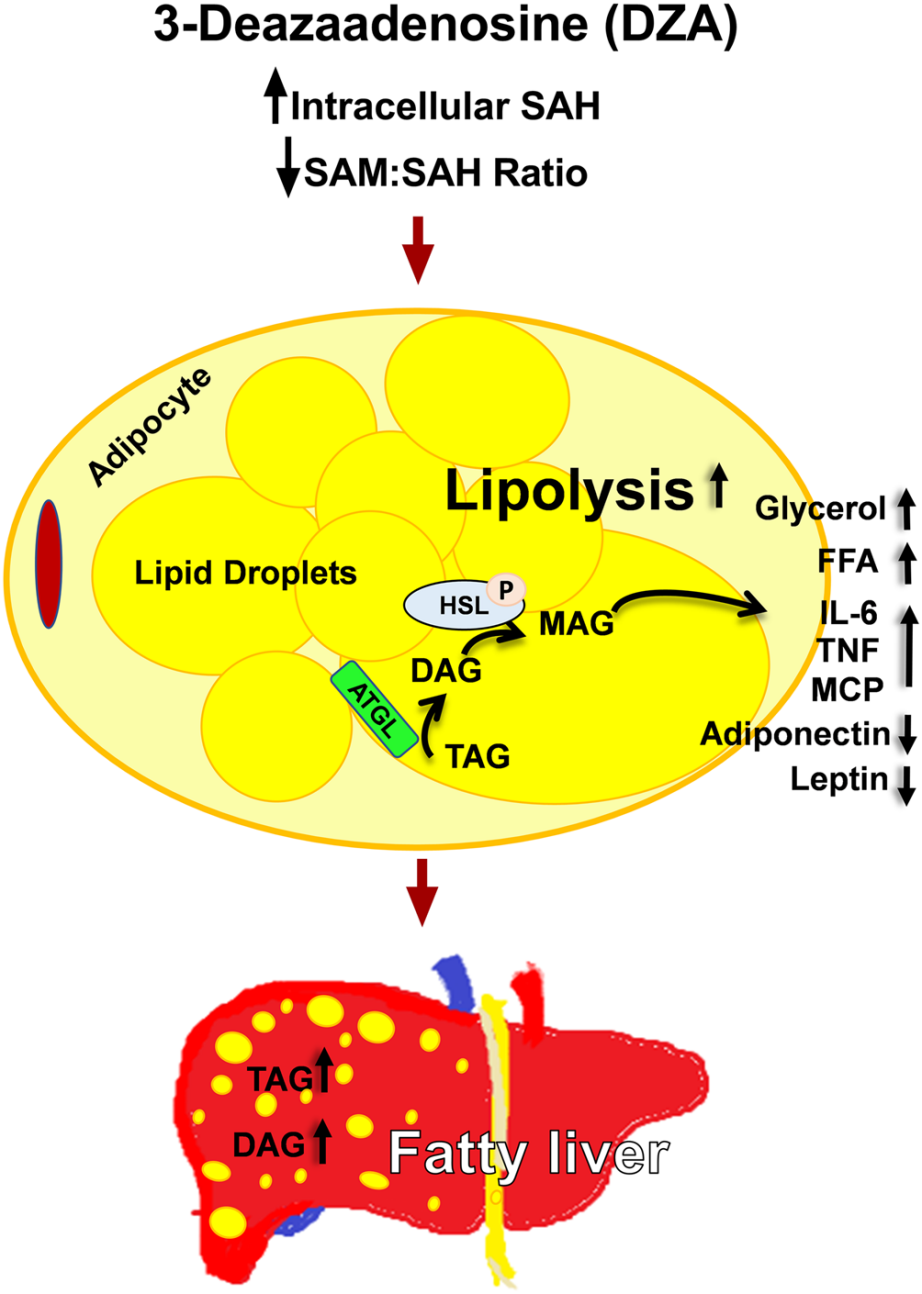
Schematic representation of the effect of DZA treatment: This study shows that the elevation in intracellular SAH levels in adipocytes by DZA treatment ultimately promotes liver injury by increasing circulating FFAs and pro-inflammatory cytokines while decreasing protective adipokine release.

The results of this study collectively demonstrate that DZA-induced increase in intracellular SAH in 3T3-L1 adipocytes (1) stimulates lipolysis by activating lipases, (2) reduces the secretion of adipokines, (3) increases the release of inflammatory cytokines and (4) downregulates adipogenesis as schematically shown in Fig. 9. To summarize, the selective elevation of intracellular SAH mimics ethanol’s effect in inducing adipose dysfunction that contributes to the pathogenesis of ALD^8, 9, 32, 33^. We conclude that alcohol-induced alterations in the adipose methionine metabolic pathway contributes to adipose dysfunction that ultimately results in the pathogenesis and progression of ALD.

## Materials and methods

### Cell culture

**Figure.10:**
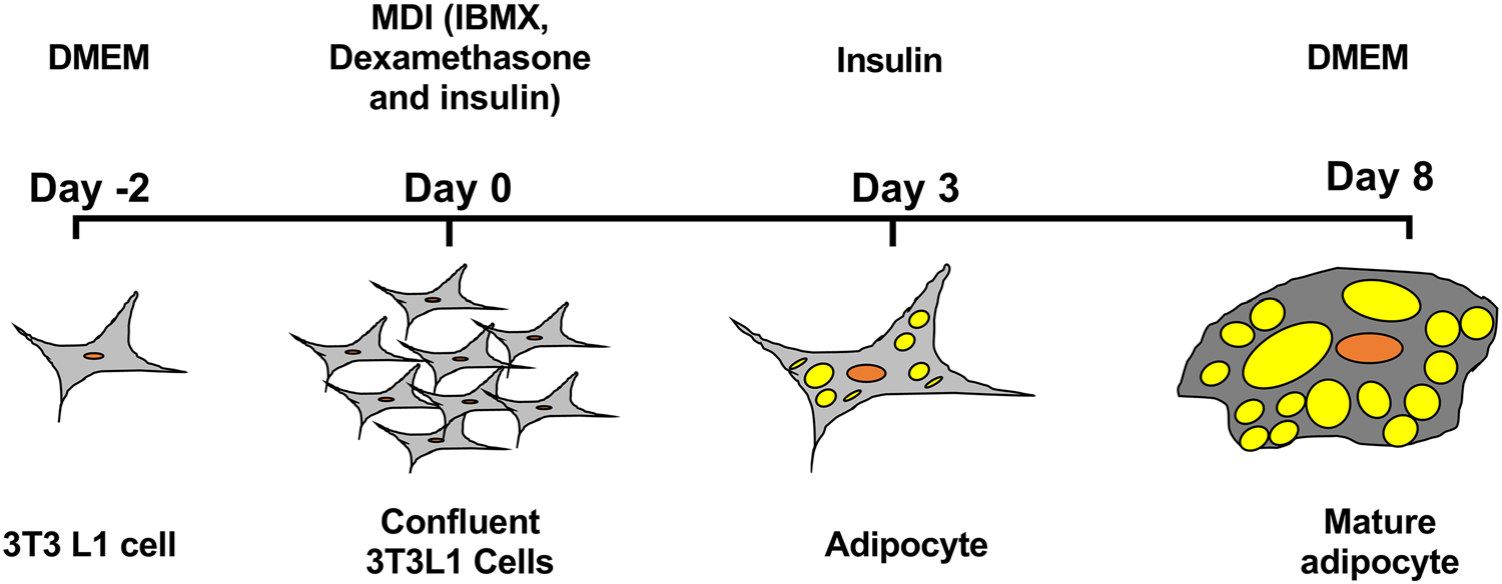
Schematic of procedure for the differentiation of 3T3-L1 pre-adipocytes into mature adipocytes.

Mouse 3T3-L1 fibroblasts were purchased from ATCC, USA. Cells were grown in DMEM medium containing 10% FBS (Hyclone Cat# SH30070.03) and 1% penicillin–streptomycin (Gibco Cat# 15240062). At 70% confluence, cells were induced to differentiate by adding methylisobutylxanthine, dexamethasone and insulin to the cultured media as described previously^76^. After three days of exposure, the differentiation medium was replaced with insulin-supplemented medium and cells cultured for the next 6 days. The cells were then maintained in 10% FBS- containing DMEM until fully differentiated into adipocytes with sufficient lipid LD formation (Fig. 10). The 3T3-L1 adipocytes were then treated with different concentrations of DZA (0 µM, 50 µM and 100 µM) for 48 h. After treatment, the spent medium was collected for measuring adiponectin, free glycerol, FFAs and adipokine levels. The cells were washed with phosphate buffered saline (PBS), harvested and lysed for further analysis.

### Estimation of triglycerides (TGs)

Total lipids were extracted from the 3T3-L1 adipocytes treated with different concentration of DZA to quantify triglyceride mass using the triglyceride diagnostics kit (Thermo DMA kit, Thermo Electron Clinical Chemistry, Louisville, CO, USA) as detailed previously^37^.

### Estimation of glycerol

The free glycerol in the culture media, which serves as an index of lipolysis, was measured using a colorimetric assay kit (Free Glycerol Reagent kit, Sigma Cat# F6428) according to the manufacturer instructions using glycerol standard solution (Sigma Cat# G7793). Glycerol levels were normalized to cellular DNA and expressed as µg glycerol/µg DNA.

### Estimation of free fatty acids (FFAs)

Levels of FFAs in the culture medium were determined by a colorimetric fatty acid detection kit (Cat# SFA-1, ZenBio Inc., Research Triangle Park, NC, USA) following the manufacturer’s instructions. Oleic acid was used as a standard. FFA concentrations were normalized to cellular DNA and expressed as µg FFA/µg DNA.

### Assay of glycerol-3-phosphate dehydrogenase (G-3-PDH) activity

G-3-PDH activity was determined based on the method by Wise and Green^77^. The fully differentiated 3T3-L1 adipocytes, grown and treated with different concentration of DZA, were rinsed twice with PBS, scraped into 300 µl of lysis buffer contain 50 mM tris-HCl (pH-7.5), 1 mM ethylenediaminetetraacetic acid, 1 mM β-mercaptoethanol and sonicated. The resulting 20 µl extract was added with 480 µl of assay buffer (100 mM triethanolamine hydrochloride (pH 7.5), 2.6 mM ethylenediaminetetraacetic acid, 0.1 mM β-mercaptoethanol, 0.12 mM nicotinamide adenine dinucleotide and 0.2 mM dihydroxyacetone phosphate) to determine the change in absorbance at 340 nm monitored at room temperature with a spectrophotometer (DU-800, Beckman Coulter, Brea, CA). 1 unit of G-3-PDH activity represents the oxidation amount of 1.0 nmol/L of substrate per minute and data are expressed as Unit/mg protein.

### BODIPY staining of adipocytes

After treatment with DZA, 3T3-L1 adipocytes were fixed in 4% w/v paraformaldehyde in 50 mM PIPES, pH 7.0, for 20 min and the accumulated lipid were visualized by staining with 1 µg/ml BODIPY 493/503 (Invitrogen, Carlsbad, CA). The cell nuclei were stained with DAPI (1 µg/ml) and were visualized using a LSM 800 confocal microscope (Carl Zeiss, Peabody, MA). Images were captured, and the staining intensity was quantified using ZEN 2.5 version software.

### Analysis of intracellular SAM and SAH

High-performance liquid chromatography (HPLC) analysis was performed on perchloric acid extracts of DZA-treated 3T3-L1 adipocytes for quantifying SAM and SAH levels to calculate the SAM:SAH ratios as detailed previously^37^.

### Immunofluorescence staining

Fully differentiated 3T3-L1 adipocytes grown on coverslips were treated with different concentrations of DZA for 48 h. The processing for immunofluorescent staining was done as detailed in our previous publication^15^. Briefly, coverslips were rinsed with PBS and fixed with 4% paraformaldehyde followed by background quenching with 50 mM NH_4_Cl in PBS. Cells were permeabilized with 0.1% Triton X-100 in PBS for 5 min at RT and blocked with 2% BSA in PBS for 1 h at RT. The coverslips were then placed into a humidified chamber and incubated for 2 h at 37 °C with 1 µg/mL each of anti-PLIN1 (Cat#3467), anti-ATGL (Cat#2138), anti-HSL (Cat#4107), anti-pHSL(Ser-563; Cat#4139), anti-pHSL (Ser-565, Cat#4137), anti-adiponectin (Cat#2789), anti-PPARγ (Cat#2435) and anti-C/EBPα (Cat#8178), anti-FABP4 (Cat#20R-2706;, anti-pATGL (Ser-406,Cat#ab135093), anti-GOS2 (Cat#ab183465) and anti-CGI-58 (Cat#ab183739) from Abcam, Cambridge, MA, USA] in PBS containing 2% BSA and 0.1% Triton X-100. All antibodies were purchased from Cell Signaling Technology (Danvers, MA, USA) except p-ATGL, GOS2, CGI-58 purchased from Abcam (Cambridge, MA, USA) and FABP4 from Fitzgerald, Acton, MA, USA). Coverslips were then washed with PBS and then incubated for 1 h at room temperature with the corresponding Alexa Fluor 647 (Cat#A21235, Invitrogen, Waltham, MA, USA) or Alexa Fluor 555 (Cat#A21422, Invitrogen, Waltham, MA, USA) diluted 1:1000 in PBS containing 2% BSA and 0.1% Triton X-100. After incubation, the coverslips were washed with PBS, stained with DAPI (1 µg/mL) and mounted. We visualized the cells under a LSM 800 confocal microscope (Carl Zeiss, Peabody, MA) for ATGL, pATGL-Ser(406), HSL, CGI-58, FABP4, adiponectin, PPARγ, C/EBPα and GOS2 expressions and Keyence BZ-X810 florescence microscope for visualizing pHSL-Ser(563), pHSL-Ser(565) and PLIN1 expressions. The staining intensity in the captured images were quantified using ZEN 2.5 version software and Keyence BZ-X810 Analyzer software, respectively.

### Adiponectin and leptin secretion

Total adiponectin and leptin levels in culture medium were determined by using specific ELISAs (R&D Systems, Inc., Minneapolis, MN, USA) according to the manufacturer’s instructions.

### Inflammatory cytokine secretion

Mouse TNF (Cat# 558534), IL-6 (Cat#555240) and MCP-1 (Cat# 555260) levels in culture media were measured by using specific ELISA kits following the manufacturer’s instructions (BD Biosciences, Pharmingen, San Diego, CA, USA).

### mRNA quantification

Total RNA was isolated from control and DZA-treated 3T3-L1 adipocytes as detailed previously^15^ using PureLink RNA Mini Kit according to the manufacturer’s instructions. The concentration of the RNA and 260/280 nm optical density (OD) ratio was determined spectrophotometrically (NanoDrop Technologies, Wilmington, DE). Two hundred ng RNA was reverse transcribed to cDNA using the high capacity reverse transcription kit. Then the cDNA was amplified using TaqMan Universal Master Mix-II with fluorescent-labeled FAM primers (TaqMan gene expression systems). After incubation in a Model 7500 qRT-PCR thermal cycler, the relative quantity of each RNA transcript was calculated by its threshold cycle (Ct) after subtracting that of the reference cDNA (β-actin). Data are expressed as the relative quantity (RQ) of transcript.

### Statistical analysis

All experimental data are expressed as mean values ± SEM. Comparisons among multiple groups were determined by one-way ANOVA, using a Tukey post-hoc test. For comparisons between two groups, we used Student’s t-test. A probability values of 0.05 or less was considered significant.

## Supplementary Information

Supplementary Information 1.

Supplementary Information 2.

Supplementary Information 3.

Supplementary Information 4.

Supplementary Information 5.

Supplementary Information 6.

Supplementary Information 7.

Supplementary Information 8.

Supplementary Information 9.

## Abbreviations

ACC: Acetyl-CoA carboxylase
ALD: Alcohol-associated liver disease
ATGL: Adipose triglyceride lipase
BODIPY: Boron-dipyrromethene
C/EBPα: CCAAT/enhancer-binding protein alpha
CD36: Cluster of differentiation 36
CGI-58: Comparative gene identification-58
DAPI: C4′,6-diamidino-2-phenylindole
DZA: 3-Deazaadenosine
GOS2: G0/G1 switch gene 2
FABP4: Fatty acid binding protein 4
FAS: Fatty acid synthase
FFA: Free-fatty acid
G-3-PDH: Glycerol-3-phosphate dehydrogenase
HSL: Hormone sensitive lipase
IL-6: Interleukin 6
LDs: Lipid droplets
mRNA: Messenger RNA
MCP-1: Monocyte chemoattractant protein-1
NEFA: Non-esterified fatty acid
PLINs: Perilipins
PPARγ: Peroxisome proliferator activated receptor gamma
RT-PCR: Reverse transcription polymerase chain reaction
SAM: S-adenosylmethionine
SAH: S-adenosylhomocysteine
TG: Triglyceride
TNF: Tumour necrosis factor
