# Supplementary material for: Elevated S-adenosylhomocysteine induces adipocyte dysfunction to promote alcohol-associated liver steatosis
Source: Sci Rep. 2021 Jul 19;11:14693. doi: 10.1038/s41598-021-94180-x (PMC8289835; doi:10.1038/s41598-021-94180-x)
Supplement: Supplementary file 1 — Supplementary Information 1. [file 41598_2021_94180_MOESM1_ESM.docx]

**Supplementary Information**

ELEVATED S-ADENOSYLHOMOCYSTEINE INDUCES ADIPOCYTE DYSFUNCTION TO PROMOTE ALCOHOL-ASSOCIATED LIVER STEATOSIS

Madan Kumar Arumugam^1,2^, Srinivas Chava^1,2^, Karuna Rasineni^1,2^, Matthew C. Paal^1,2^, Terrence M. Donohue Jr.^1-3^, Natalia A. Osna^1,2^, Kusum K. Kharbanda^1-3^**^*^**

^1^Research Service, Veterans Affairs Nebraska-Western Iowa Health Care System, Omaha, Nebraska, 68105, USA. ^2^Department of Internal Medicine and ^3^Department of Biochemistry & Molecular Biology, University of Nebraska Medical Center, Omaha, Nebraska, 68198, USA.

^*^**Corresponding author:** Kusum K. Kharbanda, Ph.D., Veterans Affairs Nebraska-Western Iowa Health Care System, Research Service (151), 4101 Woolworth Avenue, Omaha, Nebraska, 68105, USA. Tel.: 402-995-3752; Fax: 402-995-4600; E-mail address: **kkharbanda@unmc.edu.**


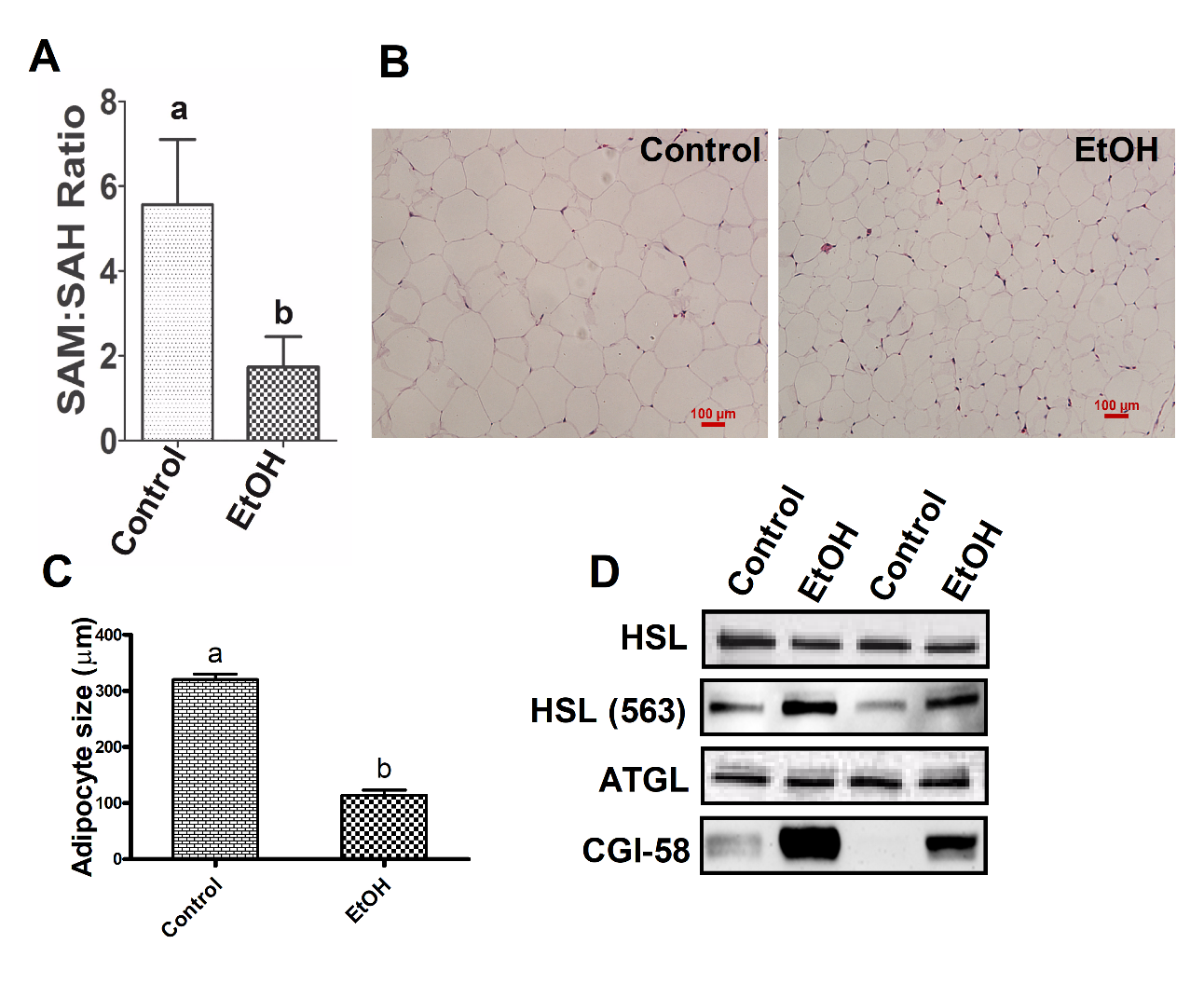


**Figure S1:** Chronic ethanol feeding alters epididymal white adipose tissue (eWAT) methylation potential, reduces adipocyte size and activates lipases. A) SAM:SAH ratio, (B) Histology images of hematoxylin and eosin stained eWAT sections (Scale bar-100µm). (C) Quantification of adipocyte size in diameter (µm) and (D) Western blot showing activation of both HSL and ATGL in eWAT of rats fed Lieber-DeCarli control or ethanol (EtOH) diets for 4-5 weeks. Data are presented as the mean ± SEM; values not sharing a common letter significantly differ from each other at p ≤ 0.05. The full-length blots/gels are presented in supplementary Figures S4- S7.


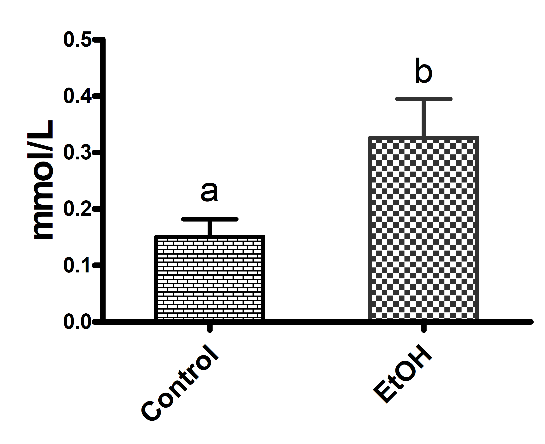


**Figure S2:** Circulating NEFA levels in rats fed the Lieber-DeCarli control or ethanol (EtOH) diets for 4-5 weeks. Data are presented as the mean ± SEM; values not sharing a common letter significantly differ from each other at p ≤ 0.05.


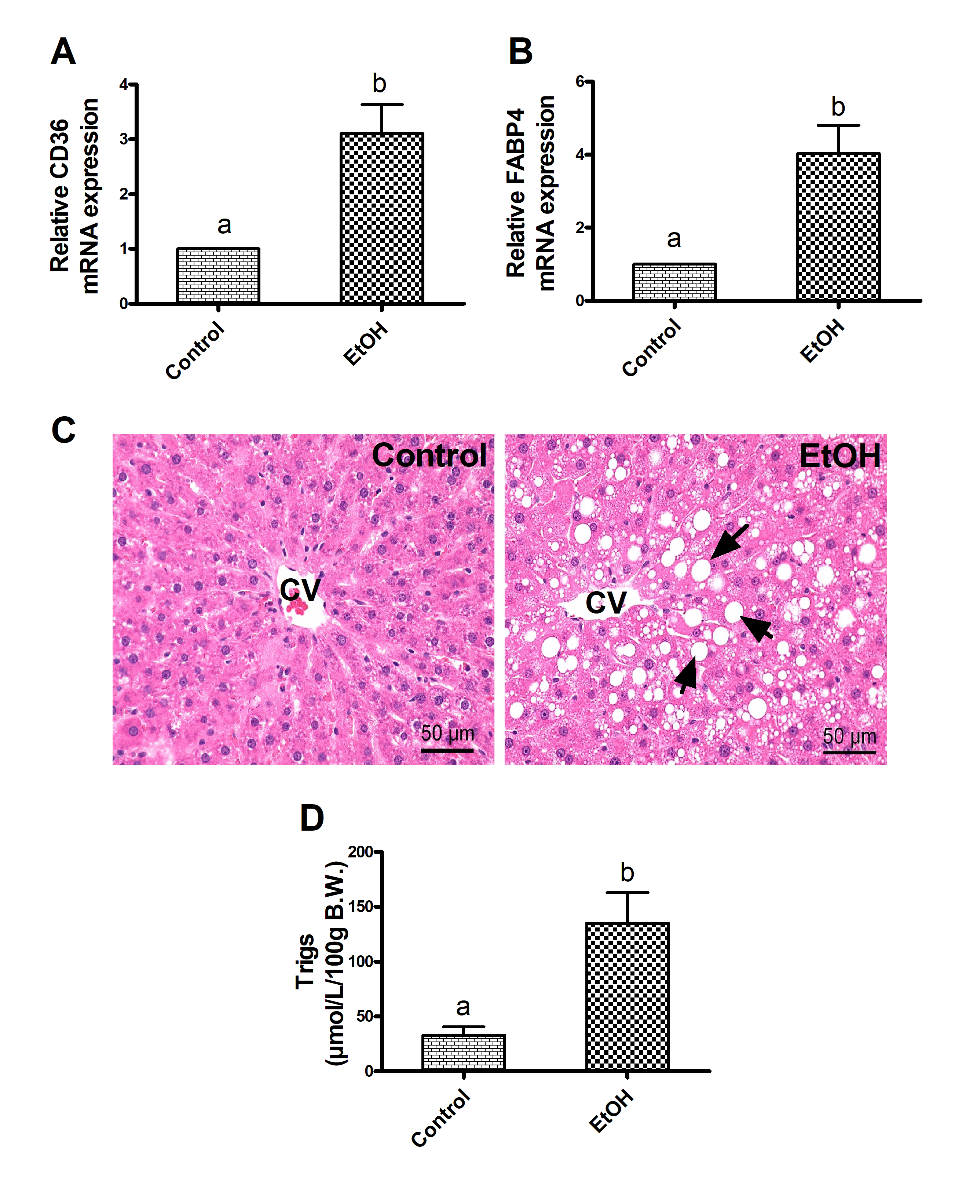


**Figure S3:** Chronic ethanol administration increases hepatic expression of fatty acid binding/ transport proteins and hepatic steatosis. Rats were fed the Lieber-DeCarli control or ethanol (EtOH) diet for 4-5 weeks, after which livers were removed and processed as detailed in the *Methods* section. (A) Relative levels of mRNAs encoding CD36 and (B) FABP4, (C) Histology images showing increased lipid droplets (arrowheads) in hematoxylin and eosin stained liver section of representative rat fed the ethanol diet (Scale bar-50µm), (D) Hepatic triglyceride (Trigs) levels. Data are presented as the mean ± SEM, (n = 6); values not sharing a common letter significantly differ from each other at p ≤ 0.05.


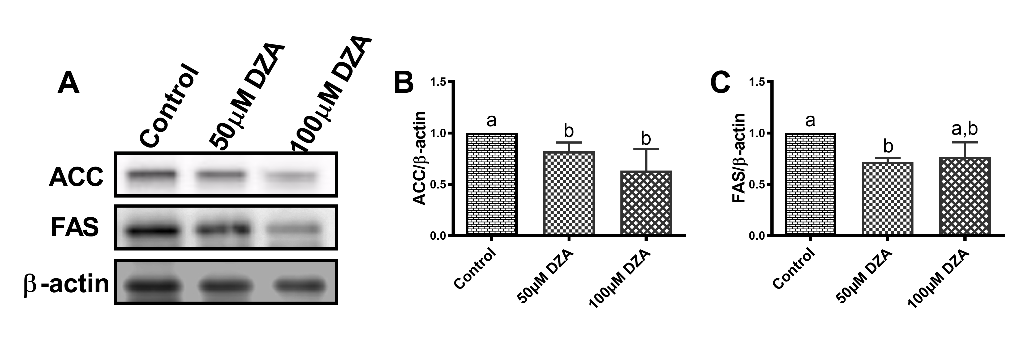


**Figure S4**. (A) Representative Western blot showing ACC and FAS levels in control and DZA-treated 3T3-L1 adipocytes. Immunoblot analysis summarizing the protein band density of (B) ACC and (C) FAS to β-actin, n = 4. Data are presented as the mean ± SEM; values not sharing a common letter significantly differ from each other at p ≤ 0.05.

**Results**

**Effects on ethanol administration on eWAT and liver**

Adipocyte size reflects the size of the unilocular lipid droplet (LD) within these cells. Histopathological assessment of eWAT revealed that chronic ethanol administration lowered the eWAT SAM:SAH ratio because of intracellular SAH increase (Fig. S1A) and reduced adipocyte LD size (Figs.S1B & S1C) compared with controls. Chronic alcohol feeding did not affect the expression of either total hormone sensitive lipase (HSL) or adipose triglyceride lipase (ATGL), but it significantly elevated the levels of phosphorylated hormone sensitive lipase (p-HSL(Ser 563)) and CGI-58, the ATGL activator (Fig. S1D). Associated with the reduction in adipocyte LD size and activation of the two lipases, we observed two-fold higher serum NEFA levels in ethanol-fed rats compared with controls (Fig. S2). Concurrent with ethanol-induced changes in eWAT, we observed significant increase in hepatic levels of mRNAs encoding fatty acid transport/binding proteins (cluster of differentiation 36 (CD36; Supplementary Fig. S3A) and adipose-specific FABP4 (Supplementary Fig. S3B)), suggesting accelerated uptake of adipose-derived circulating FFA, thereby promoting the development of hepatic steatosis. Indeed, histopathological assessment revealed marked micro- and macro-vesicular hepatic steatosis (Fig. S3C) with numerous lipid droplets around the central vein in liver sections of ethanol-fed rats. Biochemical analysis corroborated the histological assessment, as we observed 2-3-fold higher triglyceride levels in livers of ethanol-fed rats than controls (Fig. S3D).

**Effects of DZA on the *de nova* lipogenesis in 3T3-L1 adipocytes**

The level of triglycerides depicted DZA treated adipocytes as shown in Figure 1C could also be due to inhibition of de novo lipogenesis. As shown in Figure S4, DZA treatment did indeed cause a modest but significant decline in the levels of ACC and FAS proteins, reaching a maximum decrease of ~25% for each enzyme after exposure to 100 µM DZA. These data indicate that reduced lipogenesis likely contributes to the attrition of lipid droplets from these cells, but not to the degree to which lipolysis was increased.

**Materials and Methods**

Lieber-DeCarli control and ethanol liquid diets were purchased from Dyets Inc. (Bethlehem, PA). Male Wistar rats weighing 180 to 200 g obtained from Charles River Laboratories, Wilmington, MA) were weight-matched and pair-fed the Lieber-DeCarli control or ethanol liquid diets^1^ for 4-5 weeks, as described previously^2^. All animals received the care, use and procedures performed on these rats complied with NIH guidelines and all procedures were approved by the Institutional Animal Care and Use Committee at the Omaha Veterans Affairs Medical Center.

After pair-feeding for 4-5 weeks, the animals were sacrificed, blood was collected, and the epididymal white adipose tissue (eWAT) was removed. Portions of the eWAT were immediately fixed in formalin for histology or processed for the preparation of a deproteinized extract using perchloric acid for HPLC analysis of SAM and SAH Serum was prepared by centrifuging whole blood in serum separator tubes at 13,000Xg for 5 min. The remaining adipose tissue was freeze-clamped and stored at -70ºC for Western blot analysis.

***Histology:*** Formalin-fixed liver and adipose tissue sections were prepared, stained with hematoxylin and eosin and assessed for pathological changes. Digital images were acquired using a Keyence BZ-X810 microscope (Plano, TX, USA).

***SAM:SAH Ratio:*** High-performance liquid chromatography (HPLC) analysis was performed on perchloric acid extracts of eWAT for quantifying SAM and SAH levels as detailed previously^2^. SAM:SAH ratios were determined from these analyses as described previously^2^.

***Western Blot:*** Adipose tissue homogenates and 3T3-L1 adipocyte cell lysate were subjected to Western blot analysis using 1:1000 dilution of primary antibodies directed against HSL (Cat#, 4107), pHSL(Ser-563; Cat#4139), ATGL (Cat#2138), CGI-58 (Cat#ab183739), ACC (Cat# 3676) or FAS (Cat# ab22759) as previously described^2^. All antibodies were purchased from Cell Signaling Technology (Danvers, MA, USA) except CGI-58 and FAS which was purchased from Abcam (Cambridge, MA, USA). After incubation of the membranes with the appropriate secondary antibodies, proteins were visualized using standard enhanced chemiluminescence detection methods and imaged using a BIORAD ChemiDoc MP imaging system software (Bio-Rad Laboratories, Hercules, CA, USA). The intensities of immunoreactive protein bands were quantified using Quantity One software (Bio-Rad Laboratories, Hercules, CA, USA).

***Non-esterified fatty acid (NEFA):*** Serum NEFA levels were quantified using the NEFA-HR diagnostic kit from Wako Life Sciences (Mountain View, CA) as detailed^3^.

***mRNA quantification:*** Total RNA was isolated from the liver of control and ethanol treated animals as detailed previously^4^ using PureLink RNA Mini Kit according to the manufacturer’s instructions. The concentration of the RNA and 260/280 nm optical density (OD) ratio was determined spectrophotometrically (NanoDrop Technologies, Wilmington, DE). Two hundred ng RNA was reverse transcribed to cDNA using the high capacity reverse transcription kit. Then the cDNA was amplified using TaqMan Universal Master Mix-II with fluorescent-labeled FAM primers (TaqMan gene expression systems). After incubation in a Model 7500 qRT-PCR thermal cycler, the relative quantity of each RNA transcript was calculated by its threshold cycle (Ct) after subtracting that of the reference cDNA (β-actin). Data are expressed as the relative quantity (RQ) of transcript.

***Statistical Analysis:*** Data were analyzed by ANOVA followed by Tukey post-hoc test for comparisons between groups. Results were considered statistically different with p values ≤ 0.05.

**References**

1 Lieber, C. S., DeCarli, L. M. & Sorrell, M. F. Experimental methods of ethanol administration. *Hepatology* **10**, 501-510, doi:10.1002/hep.1840100417 (1989).

2 Kharbanda, K. K. *et al.* Betaine attenuates alcoholic steatosis by restoring phosphatidylcholine generation via the phosphatidylethanolamine methyltransferase pathway. *J Hepatol* **46**, 314-321, doi:10.1016/j.jhep.2006.08.024 (2007).

3 Osna, N. A. *et al.* Prolonged feeding with guanidinoacetate, a methyl group consumer, exacerbates ethanol-induced liver injury. *World J Gastroenterol* **22**, 8497-8508, doi:10.3748/wjg.v22.i38.8497 (2016).

4 Arumugam, M. K. *et al.* Role of elevated intracellular S-adenosylhomocysteine in the pathogenesis of alcohol-related liver disease. *Cells* **9**, doi:10.3390/cells9061526 (2020).

**HSL**

| Lane 1 = | Control |
| --- | --- |
| Lane 2 = | Ethanol |
| Lane 3 = | Control |
| Lane 4 = | Ethanol |


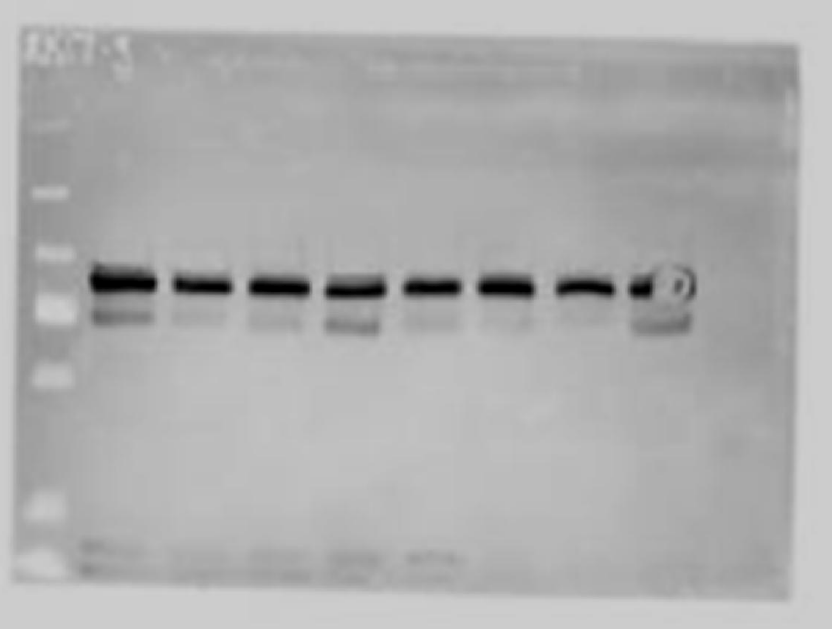


**MW**

250 kDa ---

150 kDa --

1 2 3 4

100 kDa --

50 kDa --

75 kDa --

37 kDa --

25 kDa --

20 kDa --

**Figure S5.** Unprocessed western blot image of HSL related to Figure S1D.

**pHSL-Ser(563)**

| Lane 1 = | Control |
| --- | --- |
| Lane 2 = | Ethanol |
| Lane 3 = | Control |
| Lane 4 = | Ethanol |

**MW**


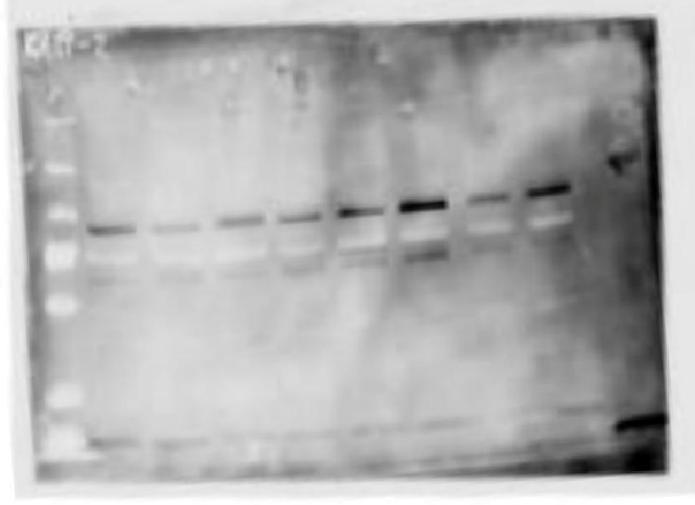


150 kDa --

250 kDa ---

1 2 3 4

100 kDa --

75 kDa --

37 kDa --

50 kDa --

25 kDa --

20 kDa --

**Figure S6.** Unprocessed western blot image of pHSL-Ser(563) related to Figure S1D.

**ATGL**

| Lane 1 = | Control |
| --- | --- |
| Lane 2 = | Ethanol |
| Lane 3 = | Control |
| Lane 4 = | Ethanol |

**MW**


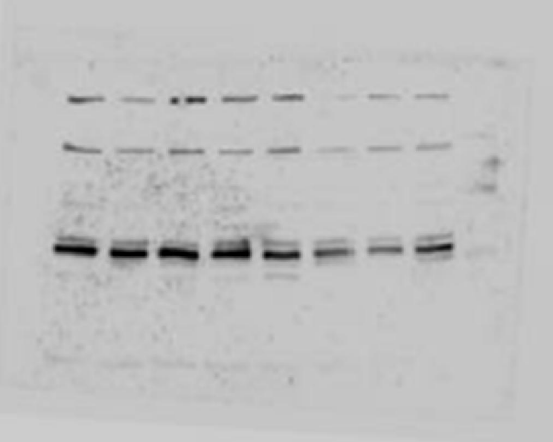


-250 kDa

-150 kDa

-75 kDa

-100 kDa

1 2 3 4

-50 kDa

-25 kDa

-37 kDa

-20 kDa

**Figure S7.** Unprocessed western blot image of ATGL related to Figure S1D.

**CGI-58**


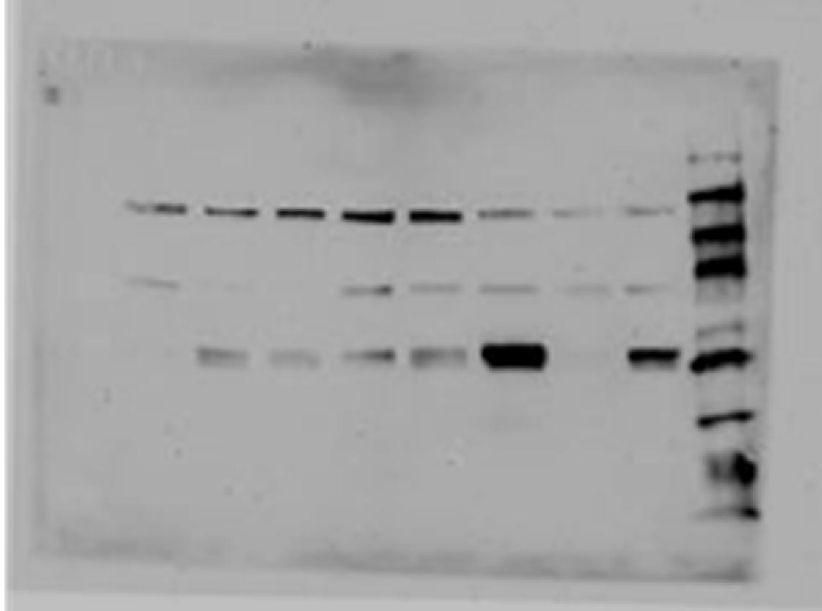


**MW**

-250 kDa

| Lane 1 = | Control |
| --- | --- |
| Lane 2 = | Ethanol |
| Lane 3 = | Control |
| Lane 4 = | Ethanol |

-150 kDa

-75 kDa

-100 kDa

1 2 3 4

-37 kDa

-50 kDa

-25 kDa

-20 kDa

-10 kDa

**Figure S8.** Unprocessed western blot image of CGI-58 related to Figure S1D.

**ACC**

**(I)** (II)

1 2 3


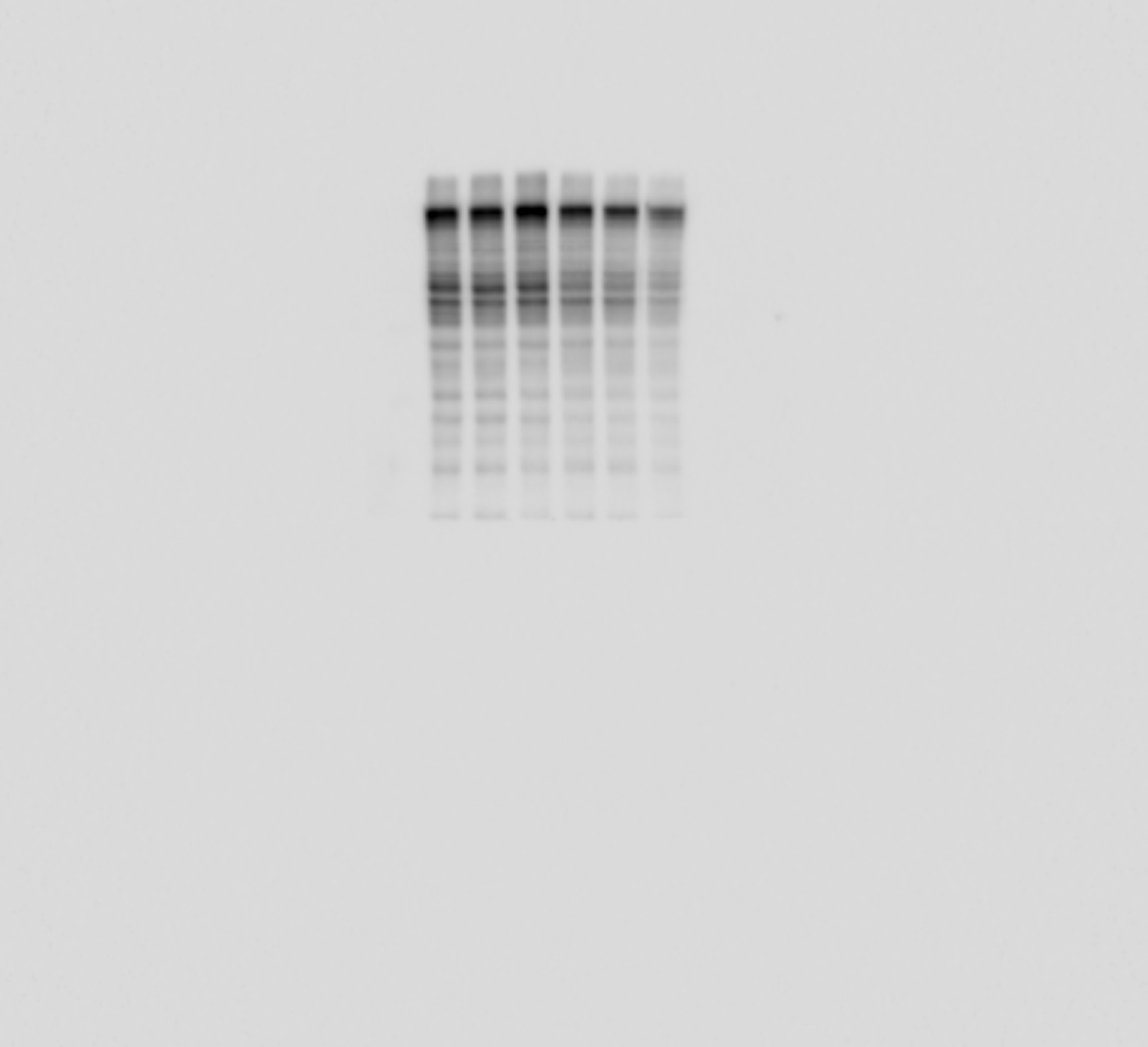

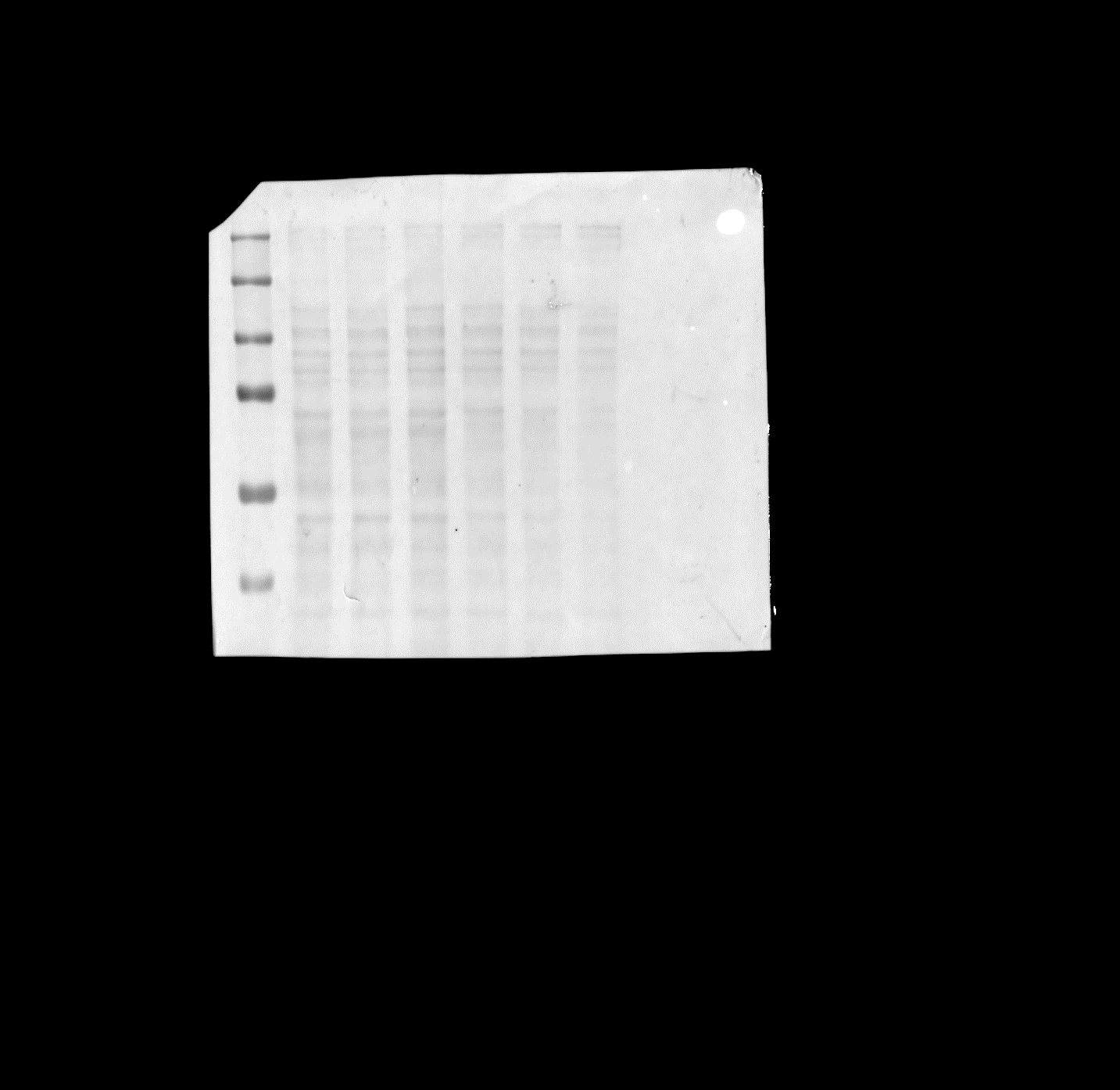


1 2 3


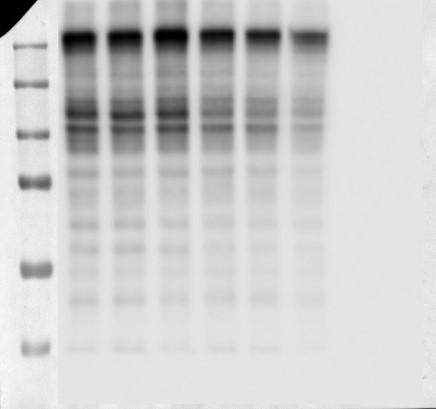
**(III)**

150 kDa --

250 kDa ---

100 kDa --

75 kDa --

| Lane 1 = | Control |
| --- | --- |
| Lane 2 = | 50µM DZA |
| Lane 3 = | 100µM DZA |

50 kDa --

37 kDa --

25 kDa --

10 kDa --

**Figure S9.** Unprocessed western blot image of ACC related to Figure S4A. (I) molecular weight markers, (II) protein blot and (III) the merged image of cropped molecular weight marker lane with the protein blot.

| Lane 1 = | Control |
| --- | --- |
| Lane 2 = | 50µM DZA |
| Lane 3 = | 100µM DZA |

**FAS**

1 2 3


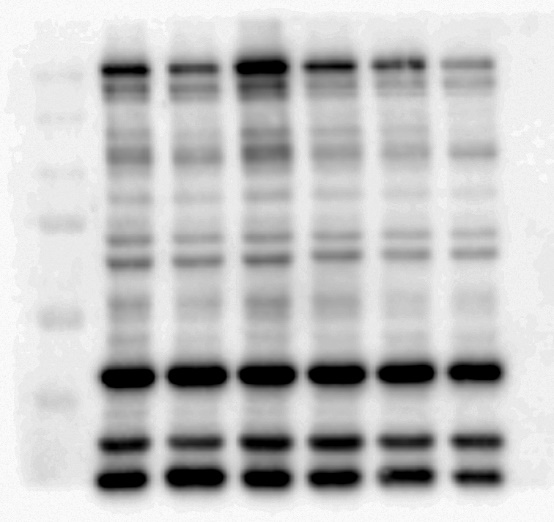


25 kDa --

10 kDa --

37 kDa --

250 kDa --

50 kDa --

75 kDa --

150 kDa --

100 kDa --

**Figure S10.** Unprocessed western blot image of FAS related to Figure S4A.

**β-Actin**

(I) (II)


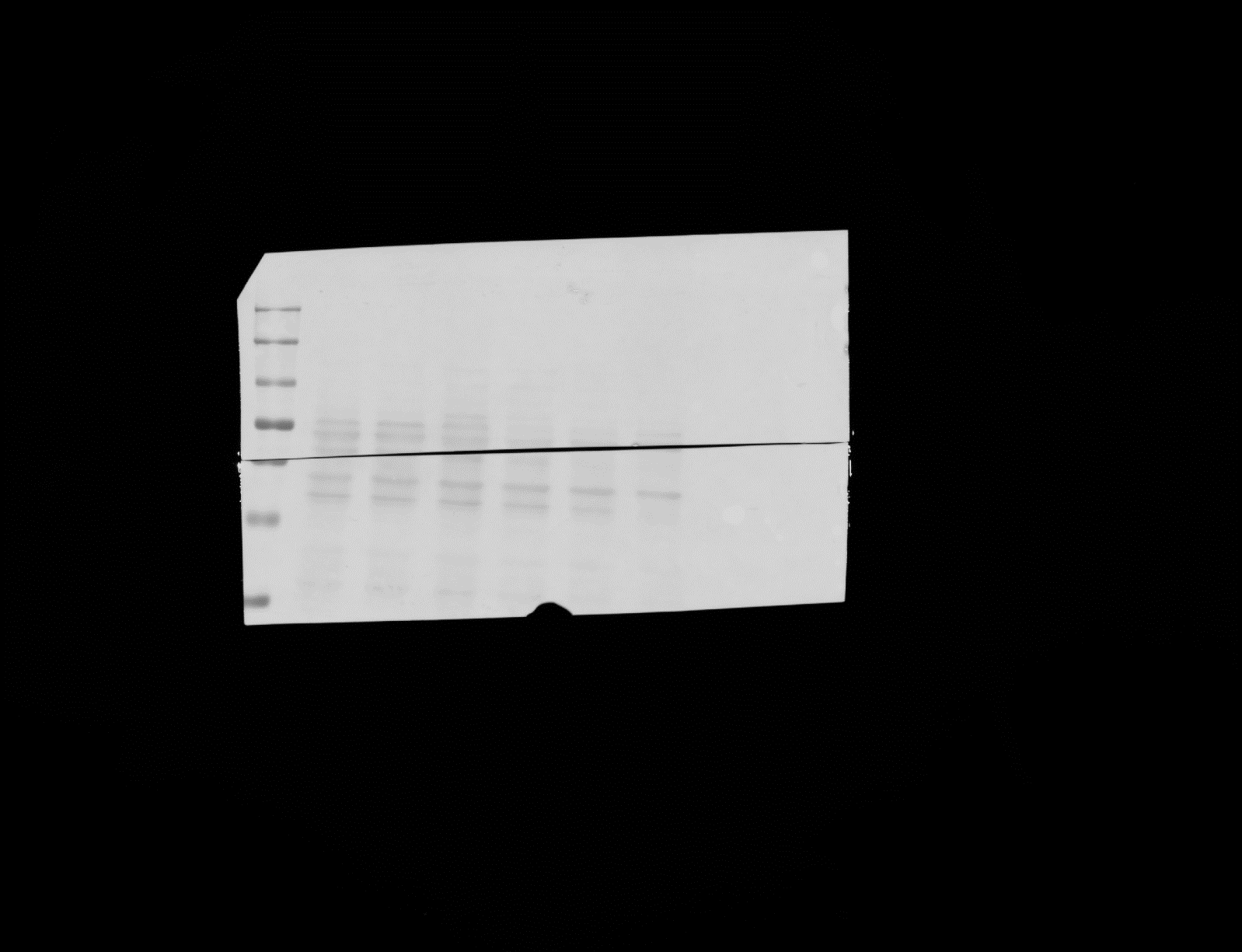

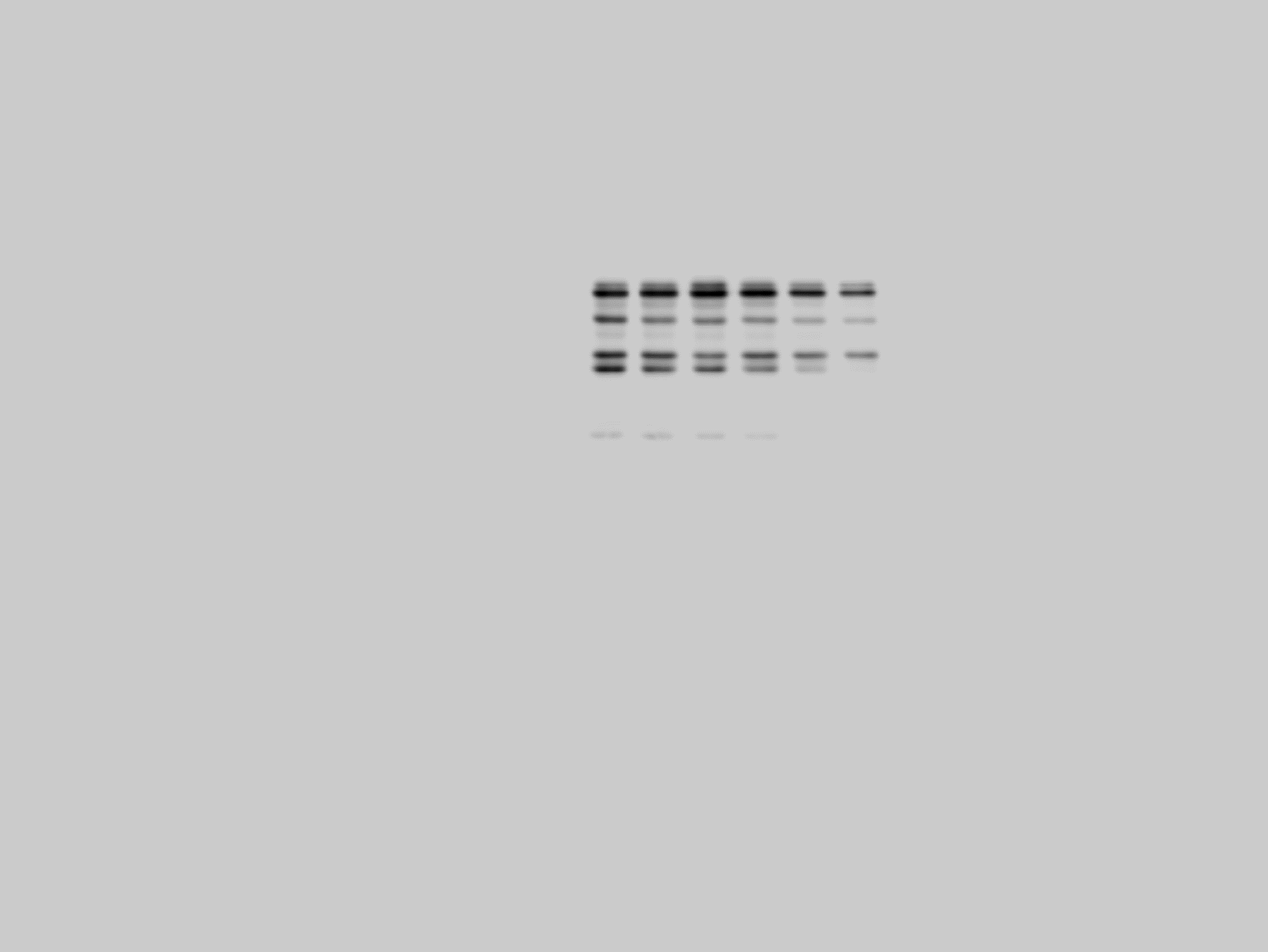


1 2 3

(III)

| Lane 1 = | Control |
| --- | --- |
| Lane 2 = | 50µM DZA |
| Lane 3 = | 100µM DZA |


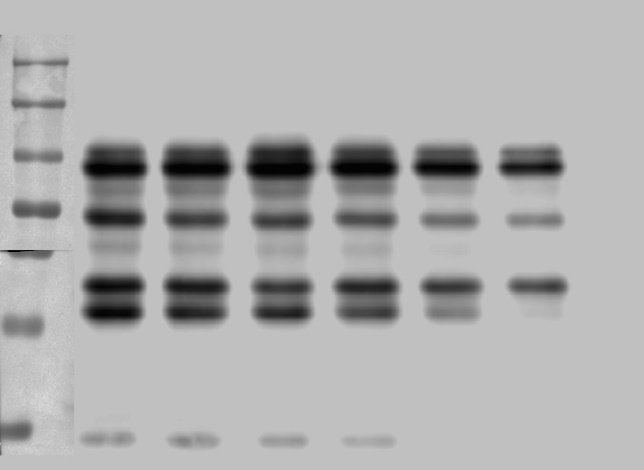


1 2 3

25 kDa --

250 kDa --

150 kDa --

100 kDa --

75 kDa --

50 kDa --

37 kDa --

10 kDa --

**Figure S11.** Unprocessed western blot image of FAS related to Figure S4A. (I) molecular weight markers, (II) protein blot and (III) the merged image of cropped molecular weight marker lane with the protein blot.
