# Supplementary figures and images for: Elevated S-adenosylhomocysteine induces adipocyte dysfunction to promote alcohol-associated liver steatosis
Source: Sci Rep. 2021 Jul 19;11:14693. doi: 10.1038/s41598-021-94180-x (PMC8289835; doi:10.1038/s41598-021-94180-x)

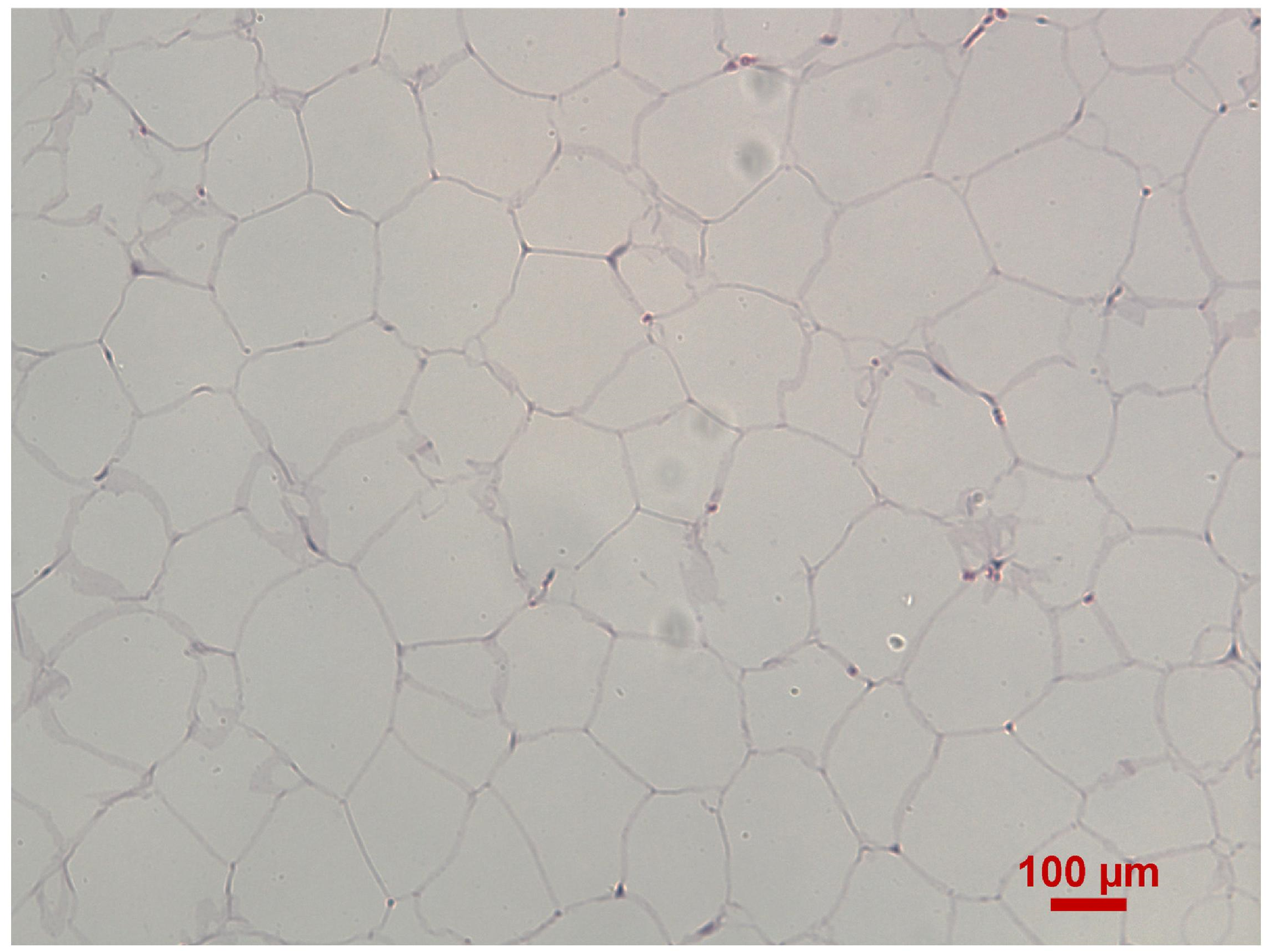

Supplement: Supplementary file 2 — Supplementary Information 2. [file 41598_2021_94180_MOESM2_ESM.tif]

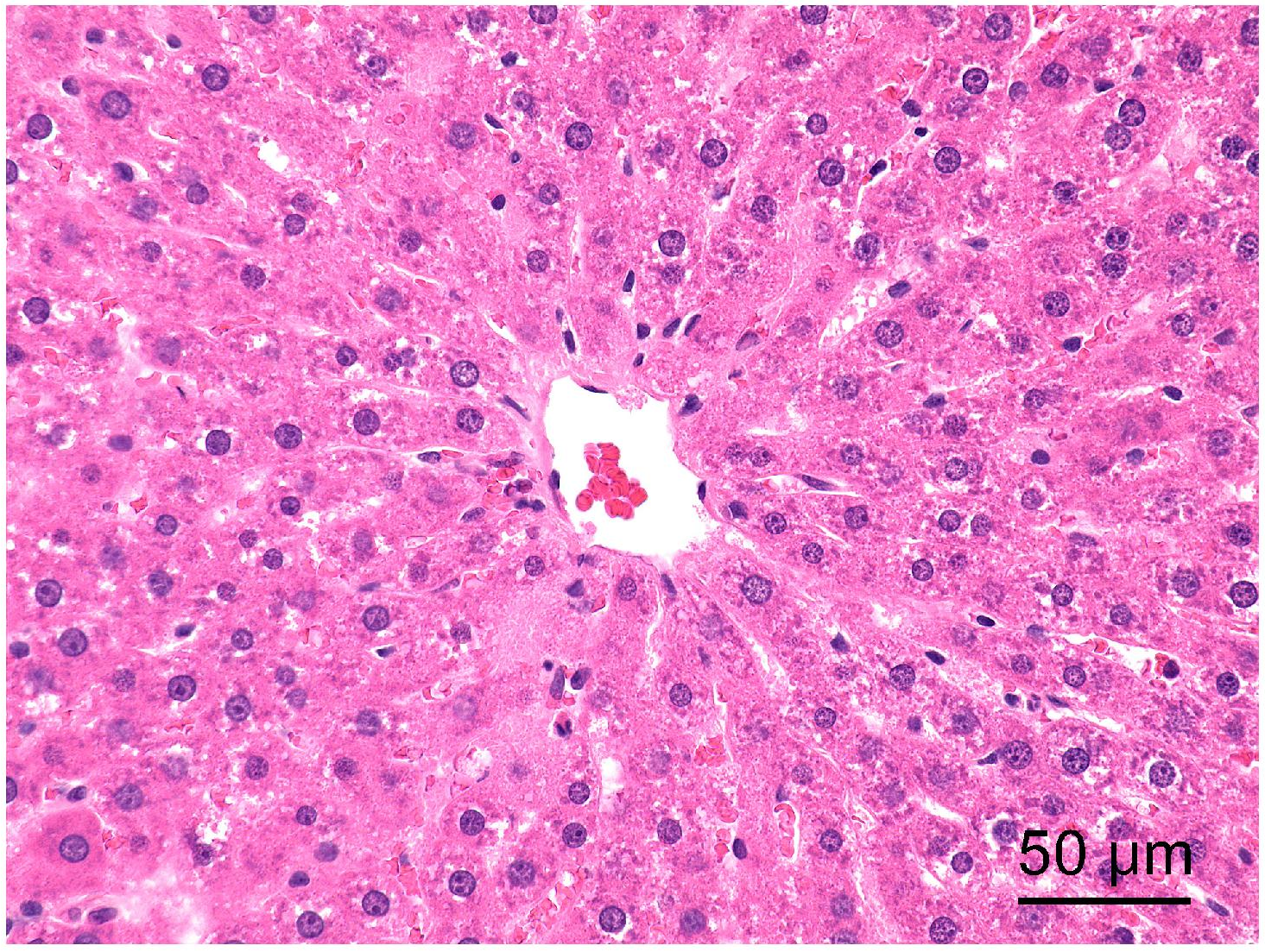

Supplement: Supplementary file 3 — Supplementary Information 3. [file 41598_2021_94180_MOESM3_ESM.tif]

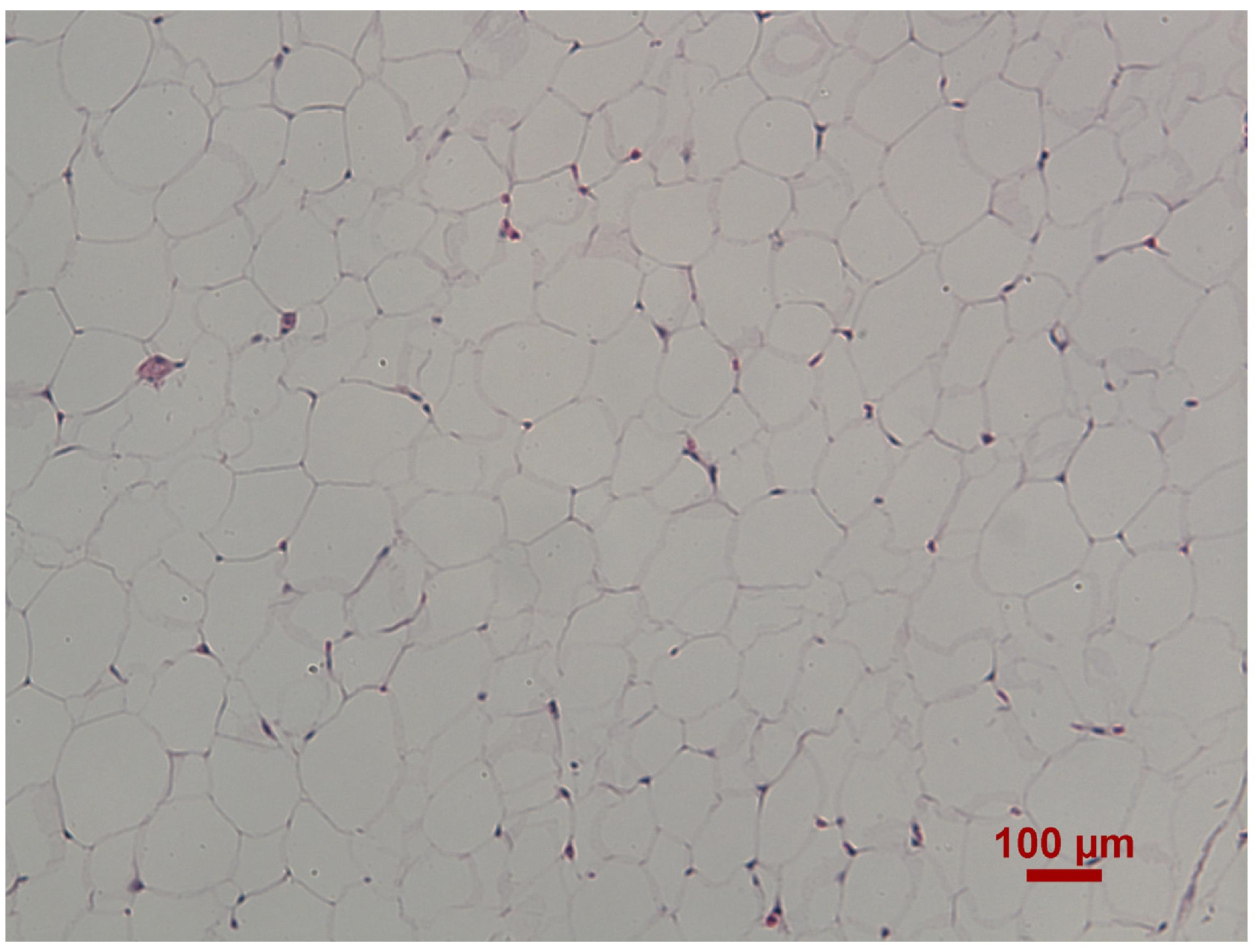

Supplement: Supplementary file 4 — Supplementary Information 4. [file 41598_2021_94180_MOESM4_ESM.tif]

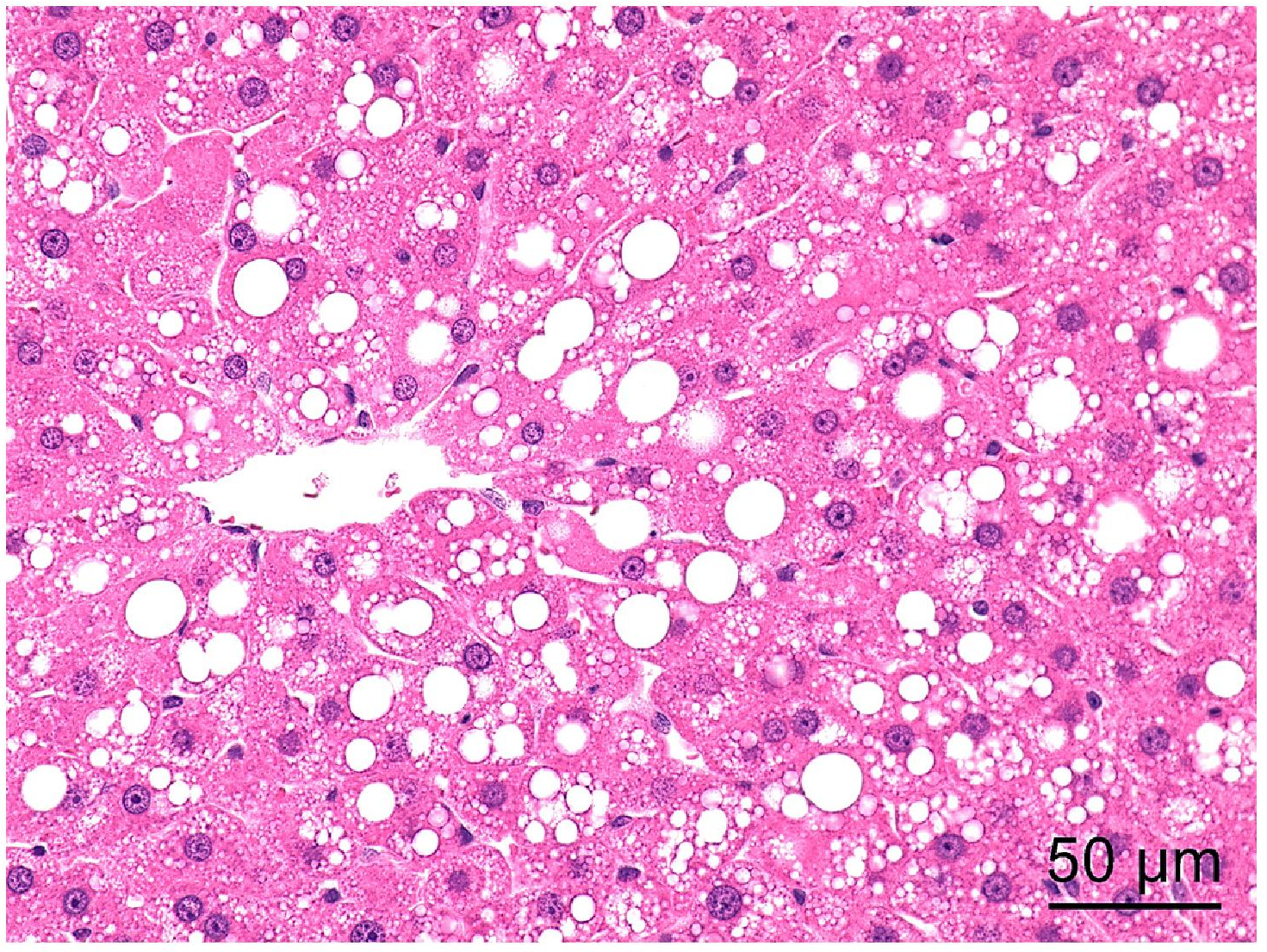

Supplement: Supplementary file 5 — Supplementary Information 5. [file 41598_2021_94180_MOESM5_ESM.tif]

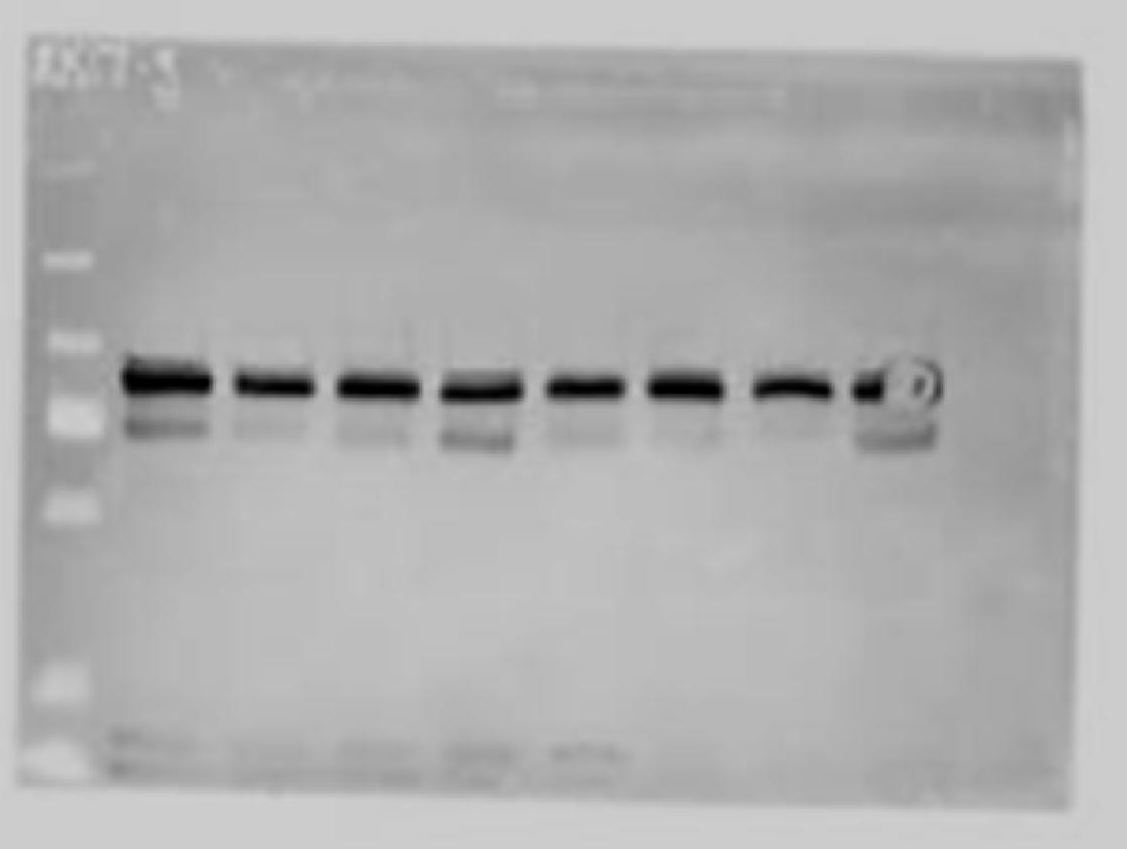

Supplement: Supplementary file 6 — Supplementary Information 6. [file 41598_2021_94180_MOESM6_ESM.tif]

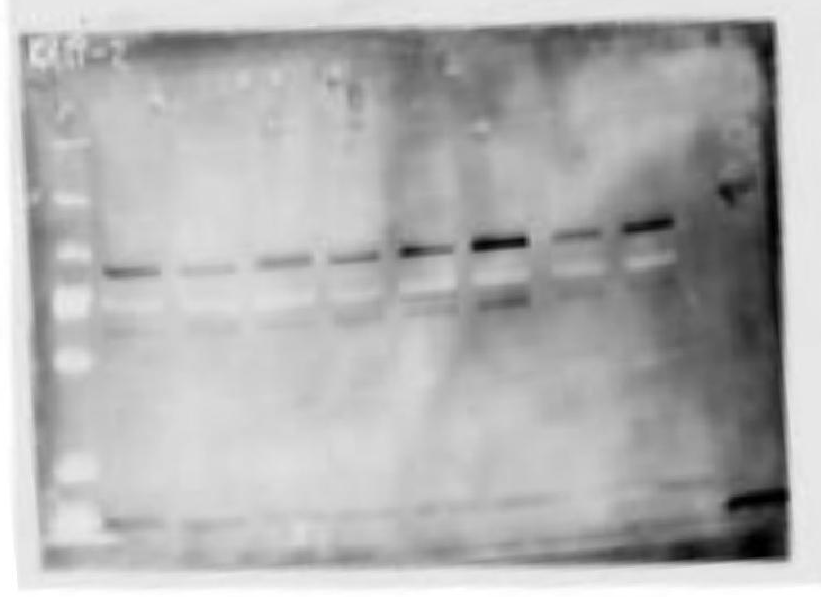

Supplement: Supplementary file 7 — Supplementary Information 7. [file 41598_2021_94180_MOESM7_ESM.tif]

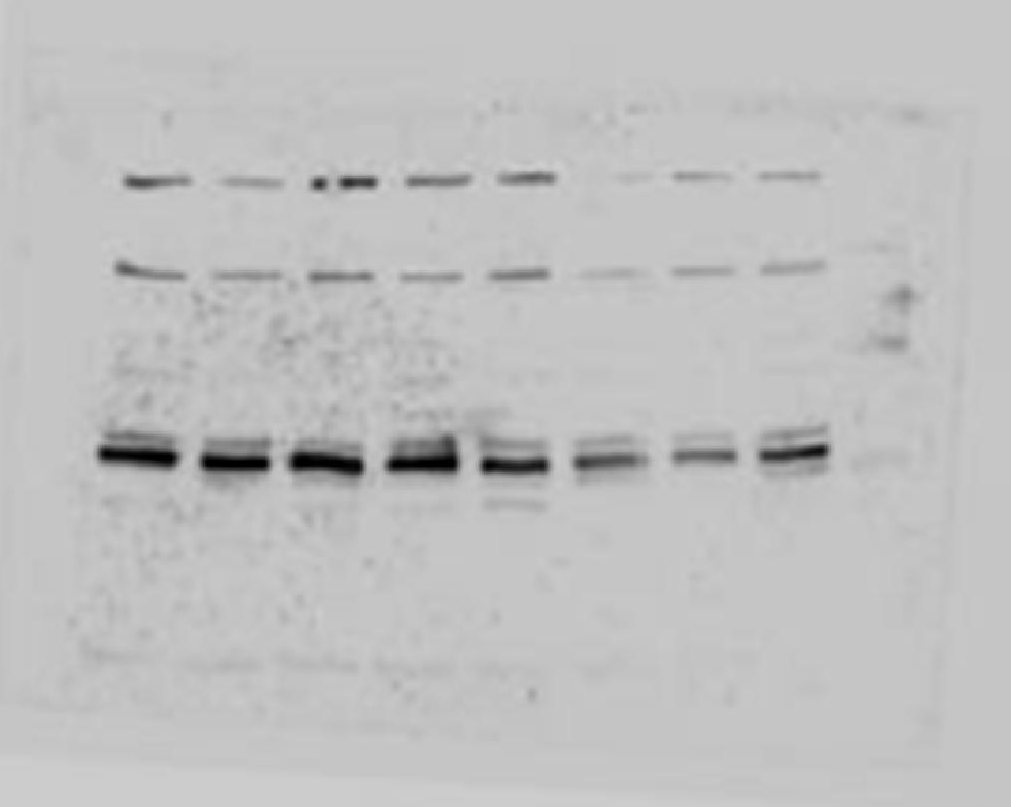

Supplement: Supplementary file 8 — Supplementary Information 8. [file 41598_2021_94180_MOESM8_ESM.tif]

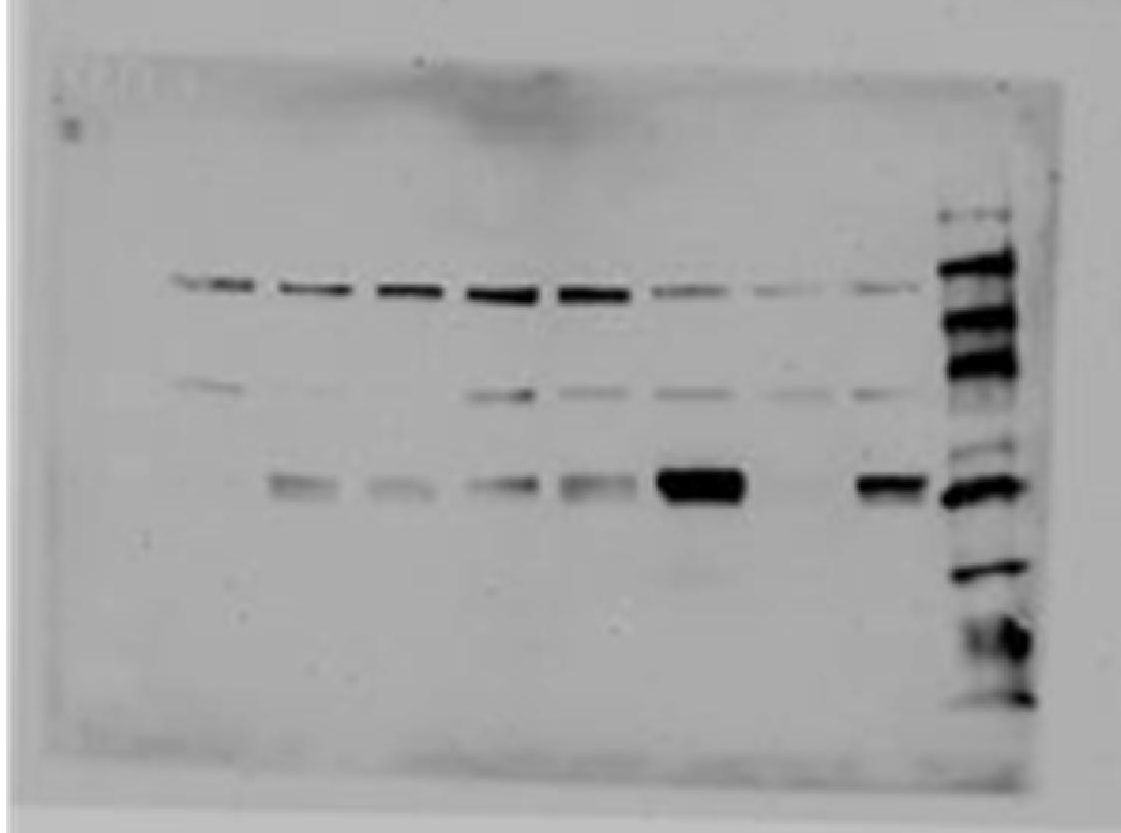

Supplement: Supplementary file 9 — Supplementary Information 9. [file 41598_2021_94180_MOESM9_ESM.tif]
